# Comparative Study on Antioxidant Potential of *Schinus terebinthifolius* Extracts Prepared by Conventional Extraction, Accelerated Solvent Extraction, and Pulsed Electric Field Method

**DOI:** 10.3390/molecules30173589

**Published:** 2025-09-02

**Authors:** Tanakarn Chaithep, Anurak Muangsanguan, Juan M. Castagnini, Francisco J. Marti-Quijal, Korawan Sringarm, Chaiwat Arjin, Pornchai Rachtanapun, Francisco J. Barba, Warintorn Ruksiriwanich

**Affiliations:** 1 Department of Pharmaceutical Sciences, Faculty of Pharmacy, Chiang Mai University, Chiang Mai 50200, Thailand; tanakarn_c@cmu.ac.th (T.C.); anurak_m@cmu.ac.th (A.M.); 2 Research Group in Innovative Technologies for Sustainable Food (ALISOST), Department of Preventive Medicine and Public Health, Food Science, Toxicology and Forensic Medicine, Faculty of Pharmacy, Universitat de València, Avda. Vicent Andrés Estellés, s/n, Burjassot, 46100 Valencia, Spain; juan.castagnini@uv.es (J.M.C.); francisco.j.marti@uv.es (F.J.M.-Q.); francisco.barba@uv.es (F.J.B.); 3 Cluster of Valorization and Bio-Green Transformation for Translation Research Innovation of Raw Materials and Products, Chiang Mai University, Chiang Mai 50200, Thailand; korawan.s@cmu.ac.th; 4 Center of Excellence in Agro Bio-Circular-Green Industry (Agro BCG), Agro-Industry, Chiang Mai University, Chiang Mai 50100, Thailand; pornchai.r@cmu.ac.th; 5 Department of Animal and Aquatic Sciences, Faculty of Agriculture, Chiang Mai University, Chiang Mai 50200, Thailand; chaiwat.arjin@cmu.ac.th; 6 School of Agro-Industry, Faculty of Agro-Industry, Chiang Mai University, Chiang Mai 50100, Thailand

**Keywords:** *Schinus terebinthifolius*, antioxidant activity, extraction methods, polyphenols, cosmeceuticals

## Abstract

Oxidative stress is a major contributor to skin aging and related disorders. This study comparatively evaluated the bioefficacy of *Schinus terebinthifolius* Raddi leaf extracts prepared using three extraction techniques: conventional extraction (CE), accelerated solvent extraction (ASE), and pulsed electric field (PEF) extraction, with 50% (*v*/*v*) ethanol and water as green solvents. Among all tested conditions, the CE-derived extract (C-4), obtained with 50% (*v*/*v*) ethanol for 120 min, exhibited the highest extraction yield (29.7%). It also showed the highest total phenolic (668.56 ± 11.52 mg gallic acid equivalent (GAE)/g dry material (DM)) and flavonoid content (2629.92 ± 112.61 mg quercetin equivalent (QE)/100 g DM), and potent antioxidant activity against 2,2′-azino-bis(3-ethylbenzothiazoline-6-sulfonic acid) (ABTS) radical (12,645.50 ± 60.31 µmol Trolox equivalent (TE)/g DM) and oxygen radical absorbance capacity assay (ORAC: 7180.27 ± 101.79 µM TE/100 g DM). Liquid Chromatography coupled with Mass Spectrometry (LC-MS) analysis revealed a diverse phytochemical profile rich in polyphenols, including gallic acid, *p*-coumaric acid, rutin, rosmarinic acid, caffeic acid, and epicatechin. Cellular assays in hydrogen peroxide (H_2_O_2_)-induced HaCaT keratinocytes demonstrated that C-4 extract significantly enhanced cell viability and upregulated endogenous antioxidant genes (superoxide dismutase (*SOD1*), catalase (*CAT*), glutathione peroxidase (*GPX*)), with effects comparable to established antioxidants such as epigallocatechin gallate (EGCG) and ascorbic acid. These findings highlight the influence of extraction parameters on phytochemical yield and biological activity, supporting the potential application of CE-derived *S. terebinthifolius* extracts as effective, sustainable ingredients for cosmeceutical formulations targeting oxidative stress-mediated skin aging.

## 1. Introduction

As the body’s largest organ, skin functions as the first line of defense against harsh environmental factors. Skin comprises nearly 16% of body weight and serves as a primary barrier against environmental hazards such as UV radiation, harmful chemicals, and pathogenic microorganisms while preventing water loss and maintaining thermal homeostasis [1]. However, the skin is continuously exposed to oxidative stress, a condition in which reactive oxygen species (ROS) exceed the skin’s natural antioxidant defenses. This can lead to premature aging, inflammation, DNA damage, impaired barrier function, increased melanin production, and eventually cell death [2,3]. To protect against oxidative damage, skin cells rely on a complex enzymatic antioxidant system. First-line defense antioxidants function to suppress or prevent the formation of free radicals and reactive species, with superoxide dismutase (SOD), catalase (CAT), and glutathione peroxidase (GPX) playing key roles. SOD initiates the detoxification process by converting superoxide radicals into H_2_O_2_. This H_2_O_2_ is subsequently broken down into water and oxygen by CAT or reduced by GPX with glutathione (GSH) serving as a cofactor. This enzyme-based defense mechanism is critical for preventing ROS production and maintaining vital cellular constituents including proteins, DNA, and lipids from oxidative damage. Disruption in the regulation or a reduction in the expression levels of these enzymes has been associated with compromised skin barrier function, premature skin aging, and increased vulnerability to oxidative stress-related conditions [4,5].

In recent years, the skincare industry has experienced a noticeable shift as consumers increasingly favor natural, antioxidant-based solutions over synthetic alternatives for preventing and treating oxidative skin damage. This growing demand has stimulated scientific interest in plant-derived antioxidants, which offer protective benefits through antioxidant and anti-inflammatory activities by modulating ROS and supporting the skin’s defense system [6]. Thailand is recognized as a rich source of medicinal plants, where traditional herbs are widely utilized in food, pharmaceutical, and cosmetic applications. For instance, in the northern region, traditional meals often include a variety of local vegetables, served raw or as side dishes. These indigenous plants are not only culturally significant but have also been recognized for their potential health benefits.

Among them, *S. terebinthifolius* Raddi (Anacardiaceae), commonly known as the Brazilian pepper, originates from Brazil, Argentina, and Paraguay but is now widely cultivated in tropical and subtropical regions, including Thailand. It is known globally as “Brazilian pepper tree”, “Florida holly”, “Christmas berry”, or “Aroeira”, and in Thailand as “Sadaobahrain” or “Matoom-Saudi”. The plant typically grows 5–10 m in height, bears small, glossy leaves, and clusters of bright red berries [7]. The dried fruits of *S. terebinthifolius* are commercially marketed as “pink peppercorns” and are widely used as culinary spices in gourmet food products [8]. It is widely consumed and has attracted considerable scientific attention due to its diverse pharmacological properties, including antioxidant, anti-cancer, wound healing, antimicrobial, and anti-inflammatory activities [9,10,11,12,13,14,15,16]. Its leaves are rich in bioactive compounds such as phenolics and essential oils. Notably, the major phenolic constituents include naringenin, gallic acid, quercetin, ferulic, and caffeic acids [17]. In traditional Brazilian medicine, preparations of the bark, leaves, and fruits have been used to treat wounds, ulcers, and inflammatory conditions [18]. Recent pharmacological and preclinical studies further support its therapeutic relevance. For instance, topical leaf oil formulations accelerated wound healing in rat models by enhancing wound contraction, collagen fiber deposition, fibroblast proliferation, and mast cell infiltration [19]. Additionally, ethyl acetate leaf extracts demonstrated significant anti-inflammatory activity when applied topically in acute skin inflammation models [20].

To preserve the bioactivity of plant compounds and support sustainable practices, green extraction techniques such as accelerated solvent extraction (ASE) and pulsed electric fields (PEF) are increasingly used. These methods employ eco-friendly solvents like water or ethanol, consume less energy, and reduce extraction time compared to conventional methods [21,22]. It is well established that different extraction methods significantly affect the yield and composition of bioactive compounds, such as phenolic compounds, which are closely associated with antioxidant activity. Therefore, a thorough comparison of extraction efficiency is essential to identify optimal techniques for maximizing the biological efficacy of plant-based products. Based on this, it is hypothesized that more efficient green extraction methods (ASE, PEF) could potentially enhance the antioxidant potential of *S. terebinthifolius* extracts by improving the recovery and bioavailability of key bioactive compounds, compared to conventional methods. Several studies have reported that ASE and PEF-assisted extractions improve the recovery of total phenolic and flavonoid contents from various plant materials [23,24].

Each extraction technique offers distinct advantages and limitations. Maceration, hereafter referred to as conventional extraction (CE), is one of the oldest and most widely used techniques. It involves soaking plant material in a suitable solvent at room temperature to facilitate diffusion-driven mass transfer of bioactive compounds. CE is advantageous due to its simplicity, low cost, and minimal equipment requirements. However, it also has several drawbacks, including long extraction times, large solvent consumption, relatively low extraction efficiency, and potential microbial contamination during extended processing. These limitations often restrict their scalability for industrial applications [25,26]. ASE has been developed to overcome some of these drawbacks by using elevated temperature and pressure to enhance solvent penetration and reduce extraction time, thereby increasing efficiency [27]. Nevertheless, ASE requires specialized instrumentation, higher operational costs, and can potentially degrade heat-sensitive compounds when high temperatures are applied [28]. PEF, in contrast, is a non-thermal technique that disrupts plant cell membranes through electroporation, facilitating the release of intracellular bioactive constitutes while minimizing thermal degradation [29,30]. Despite these advantages, PEF efficiency strongly depends on solvent polarity, conductivity, and process optimization; when applied with water, phenolic compounds are particularly prone to oxidative degradation [31,32]. A direct comparison of these methods is therefore essential to determine the most effective approach for recovering phenolic compounds from *S. terebinthifolius* leaves for potential cosmeceutical applications.

Despite the growing interest in green extraction technologies, a comprehensive comparative analysis of different extraction methods (CE, ASE, and PEF) specifically applied to *S. terebinthifolius* leaves for enhancing bioactivity has not been previously reported. The objective of this study is to compare and explore novel green extraction methods that may enhance the antioxidant capacity of *S. terebinthifolius*, with a focus on reducing oxidative stress in human keratinocytes. This study included phytochemical profiling, assessment of antioxidant capacity, and analysis of the expression of gene encoding key antioxidant enzymes (*SOD1*, *CAT*, and *GPX*). Thereby, we can determine which extraction technique yields the highest content of bioactive compounds in *S. terebinthifolius* extracts, enabling their potential use as antioxidant agents in cosmeceutical products.

## 2. Results

### 2.1. Yield of Extracts

Extract codes were systematically assigned based on the extraction method, solvent type, temperature, and extraction time. “C”, “A”, and “P” refer to CE, ASE, and PEF-assisted extraction, respectively. CE samples (C-1 to C-4) were extracted at 25 °C using water (C-1, C-2) or 50% (*v*/*v*) ethanol (C-3, C-4) for 15 or 120 min. ASE extracts (A-1 to A-4) were extracted at 40 °C or 120 °C under constant pressure for around 25 min. A-1 and A-2 used water, while A-3 and A-4 used 50% (*v*/*v*) ethanol at the same respective temperatures. PEF-assisted extracts (P-1 to P-4) were extracted using water (P-1, P-2) or 50% (*v*/*v*) ethanol (P-3, P-4) at 25 °C for 15 or 120 min. Extraction codes, conditions, and results are summarized in Table 1. Extraction yields were obtained from three independent replicates for each condition and are expressed as mean ± SD. Statistical analysis was performed using one-way ANOVA followed by Tukey’s post hoc test (*p* < 0.05) to determine significant differences among conditions. Overall, the extraction yields varied significantly depending on the method, solvent, temperature, and extraction time. Among the twelve extraction conditions tested, the highest yield was obtained from C-4 (conventional extraction with 50% (*v*/*v*) ethanol, 120 min), which yielded 29.7%, followed by C-3 (50% (*v*/*v*) ethanol, 15 min) with 27.4%. For water-based extractions, C-2 (conventional extraction, 120 min) yielded 17.8%, slightly higher than C-1 (15 min) at 16.2%. ASE water-based samples yielded 13.5% (A-1) and 14.7% (A-2), while ethanol-based ASE samples yielded 24.1% (A-3) and 25.6% (A-4). PEF-assisted extractions showed the lowest yields overall, with P-1 and P-2 (water) yielding 8.3% and 10.0%, and P-3 and P-4 (50% (*v*/*v*) ethanol) yielding 11.7% and 15.0%, respectively.

### 2.2. Quantitative Measurement of Total Phenolic and Total Flavonoid Content

The total phenolic content (TPC) and total flavonoid content (TFC) of Brazilian pepper extracts varied significantly depending on the extraction method, solvent type, temperature, and extraction time. The results are summarized in Table 1. The extraction method significantly affected TPC and TFC of Brazilian pepper extracts. Overall, among the three techniques, CE yielded the highest TPC and TFC with C-4 (50% (*v*/*v*) ethanol, 120 min), showing the maximum values (668.56 ± 11.52 mg GAE/g DM and 2629.92 ± 112.61 mg QE/100 g DM, respectively), followed by C-3 (15 min) with slightly lower values. ASE provided moderate yields, especially A-4 (50% ethanol, 120 °C), which presented TPC of 166.01 ± 1.83 mg GAE/g DM and TFC of 543.12 ± 6.13 mg QE/100 g DM. In contrast, PEF-assisted extraction gave the lowest values, particularly in water-based samples such as P-2, which showed a TPC of only 8.81 ± 0.11 mg GAE/g DM and no detectable TFC. The type of solvent also played a key role. Across all methods, 50% (*v/v*) ethanol consistently gave better results than water. For example, in CE, C-2 (water, 120 min) showed moderate values of TPC (246.13 ± 12.31 mg GAE/g DM) and TFC (409.01 ± 10.23 mg QE/100 g DM) but still much lower than C-4, which used 50% (*v/v*) ethanol under the same conditions. Similarly, in ASE, ethanol-based A-3 and A-4 yielded higher phenolic and flavonoid contents than water-based A-1 and A-2. A-1 (water, 40 °C), for example, had TPC of 52.11 ± 3.50 mg GAE/g DM and TFC of 61.17 ± 2.05 mg QE/100 g DM, which were significantly lower than ethanol-based samples. Temperature had a noticeable effect on extraction, especially in ASE. A-4 (120 °C) yielded higher TPC and TFC compared to A-3 (40 °C), which showed TPC of 132.09 ± 5.54 mg GAE/g DM and TFC of 474.43 ± 12.38 mg QE/100 g DM, demonstrating the benefit of elevated temperature on extraction efficiency. In contrast, for extractions conducted at room temperature (25 °C), such as CE and PEF, the solvent type and extraction time played a more important role. Extraction time also influenced the yield. In general, longer durations (120 min) gave higher TPC and TFC than shorter ones (15 min), particularly in CE and PEF. For instance, C-4 (120 min) had much higher values than C-3 (15 min) despite using the same solvent and temperature. Similarly, in PEF, P-4 (ethanol, 120 min) yielded TPC of 58.47 ± 3.72 mg GAE/g DM, while P-3 (ethanol, 15 min) had a higher value (125.09 ± 2.81 mg GAE/g DM).

### 2.3. Antioxidant Capacity

The Brazilian pepper extracts were evaluated using ABTS and ORAC assays, and the results are summarized in Table 1. Among the extraction methods, CE produced the highest antioxidant activity. Specifically, C-4 (50% (*v*/*v*) ethanol, 120 min) exhibited the strongest activity with ABTS of 12,645.50 ± 60.31 µmol TE/g DM and ORAC of 7180.27 ± 101.79 µM TE/100 g DM, followed by C-3 (15 min), which also showed high antioxidant values. ASE resulted in moderate antioxidant levels. Notably, A-4 (50% (*v*/*v*) ethanol, 120 °C) showed ABTS of 3613.60 ± 18.09 µmol TE/g DM and ORAC of 6770.08 ± 43.73 µM TE/100 g DM, slightly higher than A-3 (40 °C), with ABTS of 2699.54 ± 12.06 µmol TE/g DM and ORAC of 6758.86 ± 130.26 µM TE/100 g DM. Water-based ASE extracts such as A-2 (120 °C) exhibited lower values of ABTS of 2489.02 ± 6.64 µmol TE/g DM and ORAC of 4890.34 ± 330.55 µM TE/100 g DM compared to their ethanol samples, while A-1 (40 °C) showed the weakest activity in this group with ABTS of 639.67 ± 12.31 µmol TE/g DM and ORAC of 2423.08 ± 175.37 µM TE/100 g DM. In contrast, PEF-assisted extraction resulted in the lowest antioxidant capacity, particularly in water-based samples. For instance, P-2 (water, 120 min) and P-1 (water, 15 min) showed ABTS values of 1062.28 ± 51.60 µmol TE/g DM and 1151.60 ± 6.36 µmol TE/g DM, and ORAC values of 927.87 ± 6.46 µM TE/100 g DM and 642.34 ± 47.76 µM TE/100 g DM, respectively. Ethanol-based PEF samples (P-3 and P-4) showed slightly better antioxidant activity, but their activity was still much lower than CE and ASE. P-3 (ethanol, 15 min) had ABTS of 1616.93 ± 53.48 µmol TE/g DM and ORAC of 2489.13 ± 211.48, while P-4 (120 min) showed ABTS of 1543.30 ± 32.80 µmol TE/g DM and ORAC of 2717.73 ± 26.60 µM TE/100 g DM. The type of solvent also played a crucial role in antioxidant capacity. Across all extraction methods, extracts obtained with 50% (*v*/*v*) ethanol consistently exhibited higher ABTS and ORAC values than those extracted with water. For example, in CE, C-2 (water, 120 min) had ABTS of 2983.94 ± 50.71 µmol TE/g DM and ORAC of 2873.19 ± 59.98 µM TE/100 g DM, which were significantly lower than those of C-4, even though both used the same extraction time and temperature. A similar pattern was observed in ASE and PEF. Temperature was another important factor. In ASE, increasing the temperature from 40 °C (A-3) to 120 °C (A-4) resulted in higher ABTS and ORAC values, indicating enhanced radical scavenging activity under thermal conditions. In contrast, extractions at room temperature (25 °C), such as in CE and PEF, depended more on the solvent used and the extraction time. Lastly, extraction time influenced antioxidant outcomes, particularly in CE and PEF. In CE, C-4 (120 min) yielded higher ABTS and ORAC values than C-3 (15 min), indicating that longer durations improved antioxidant extraction. However, in PEF, the trend was less consistent. P-3 (ethanol, 15 min) exhibited higher ABTS 1616.93 ± 53.48 µmol TE/g DM than P-4 1543.30 ± 32.80 µmol TE/g DM despite the shorter time.

### 2.4. Identification of Constituents by Liquid Chromatography Mass Spectrometry (LC-MS)

Samples A-3, A-4, C-3, and C-4 were selected for LC-MS analysis due to their high antioxidant activity, enabling the identification of key bioactive compounds responsible for the detected biological effects. As shown in Table 2, The LC-MS analysis identified several phenolic acids and flavonoid compounds in the selected extracts (A-3, A-4, C-3, and C-4). Among the tested extracts, C-4 exhibited the highest total amount of phenolic compounds. C-4 extract also had the highest chemical diversity, with all six targeted compounds detected, while C-3 and A-4 extracts contained five compounds. A-3 extract had the lowest diversity with only four detected compounds. Gallic acid was the most abundant phenolic in all samples, with C-3 extract (1.618 ± 0.032 mg/g) and A-4 extract (1.542 ± 0.127 mg/g) showing the highest levels. In contrast, p-coumaric acid was detected in all except the C-3 extract, with the C-4 extract presenting the highest content (0.588 ± 0.008 mg/g). Caffeic acid and epicatechin, though generally present at lower levels, were also predominantly found in C-3 and C-4 extracts. Representative TICs and mass spectra of the selected extracts are shown in the Supplementary Materials.

### 2.5. Cytotoxic Effect of Brazilian Pepper Extracts

**Figure 1:**
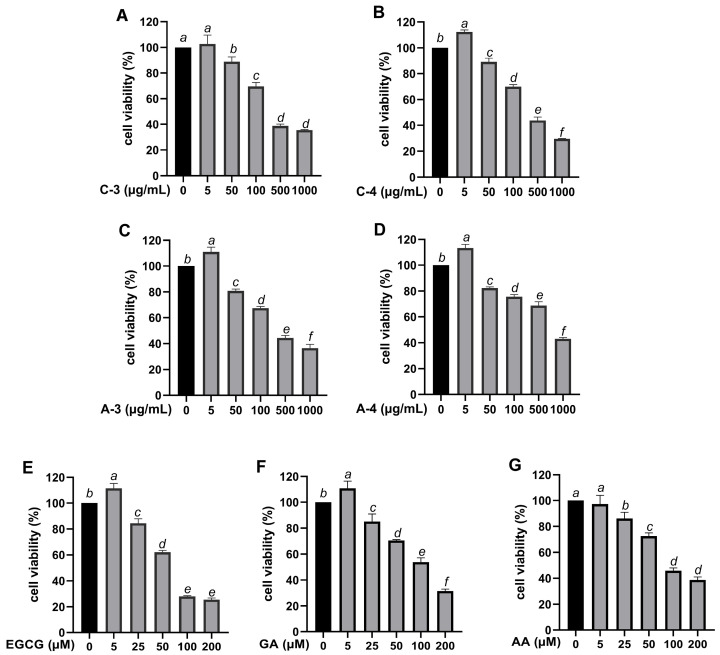
Cytotoxic effects of Brazilian pepper leaf extracts and standard antioxidants on HaCaT cells assessed by the SRB assay. (**A**–**D**) Cell viability after treatment with extracts C-3, C-4, A-3, and A-4 at concentrations of 0, 5, 50, 100, 500, and 1000 µg/mL. (**E**–**G**) Cell viability after treatment with epigallocatechin gallate (EGCG), gallic acid (GA), and ascorbic acid (AA) at concentrations of 0, 5, 25, 50, 100, and 200 µM. All values are expressed as mean ± SD (*n* = 3). Untreated cells served as the control. Statistical analyses were conducted using one-way ANOVA followed by Tukey’s post hoc test. Different letters above the bars indicate statistically significant differences among groups (*p* < 0.05).

The cytotoxicity of the samples was evaluated on HaCaT cells using sulforhodamine (SRB) assay, which quantifies cell density by measuring total cellular protein content. This method was selected for its high sensitivity, reproducibility, and broad linear range, allowing accurate detection of changes in cell viability following treatment [33]. Cytotoxicity was assessed across all concentrations ranging from 5 to 1000 µg/mL. All samples exhibited a dose-dependent reduction in cell viability, as illustrated in Figure 1. At low concentrations (5 µg/mL), C-4, A-3, A-4, EGCG, and GA significantly increased HaCaT viability above control levels (*p* < 0.05). Cell viability values slightly exceeding 100% were occasionally observed, which may occur in cell-based assays and are often interpreted as mild proliferative or stimulatory responses to the treatments, suggesting a potential cytoprotective effect. However, at higher concentrations, all extracts and standards showed a dose-dependent decrease in viability. At a concentration of 50 µg/mL, all extracts maintained over 80% cell viability, indicating no significant cytotoxicity, while EGCG, GA, and AA maintained similar low cytotoxicity at concentrations up to 25 µM. Based on these findings, 50 µg/mL for all extracts and 25 µM for EGCG, GA, and AA were considered non-cytotoxic and were used for further experiments.

### 2.6. Effect of Brazilian Pepper Extracts on Cell Viability of HaCaT Cells Under Oxidative Stress

**Figure 2:**
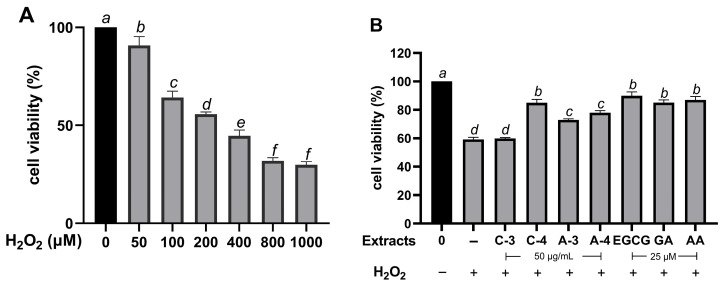
Cytoprotective effects of Brazilian pepper leaf extracts against hydrogen peroxide (H_2_O_2_)-induced oxidative stress in HaCaT cells. (**A**) Cell viability of HaCaT cells treated with increasing concentrations of H_2_O_2_ (100–1000 µM) for 24 h. (**B**) Protective effects of extract pre-treatment (50 µg/mL) and epigallocatechin gallate (EGCG), gallic acid (GA), and ascorbic acid (AA) (25 µM) prior to exposure to 200 µM H_2_O_2_. Data are presented as mean ± SD (*n* = 3). Statistical analysis was performed using one-way ANOVA followed by Tukey’s post hoc test. Different letters above the bars indicate statistically significant differences among groups (*p* < 0.05).

To establish a suitable oxidative stress model, HaCaT cells were treated with H_2_O_2_ at various concentrations ranging from 50 to 1000 µM. As shown in Figure 2A, treatment with 200 µM H_2_O_2_ reduced cell viability to approximately 50% compared with the untreated control. The concentration for subsequent experiments was carefully chosen to evaluate the extracts’ cytoprotective effects. Pre-treatment with the extracts at 50 µg/mL and the reference standard at 25 µM significantly improved cell viability (*p* < 0.05). Specifically, cell viability recovered to 84.92 ± 2.50% for C-4 extract, 78.02 ± 1.44% for A-4 extract, 72.95 ± 0.82% for A-3, and 59.98 ± 0.58% for C-3. Similarly, standard antioxidants like EGCG (89.98 ± 2.63%), GA (85.01 ± 2.06%), and AA (87.05 ± 2.43%) also restore cell viability to levels comparable to the untreated control group (Figure 2B). These findings strongly suggest that the extracts provide protective effects against H_2_O_2_-induced oxidative stress in HaCaT cells.

### 2.7. SOD1, GPX, and CAT Expression

Treatment with 200 µM H_2_O_2_ for 24 h significantly reduced HaCaT cell viability to approximately 50%, indicating effective induction of oxidative stress for the analysis of antioxidant-related genes SOD1, CAT, and GPX in the presence of Brazilian pepper extracts. Although intracellular ROS levels were not directly measured in this study, the significant downregulation of these key antioxidant genes following H_2_O_2_ treatment serves as an indirect indicator of oxidative stress. This observation aligns with previous reports showing similar gene expression patterns under oxidative insult in HaCaT cells treated with comparable H_2_O_2_ concentrations [34].

**Figure 3:**
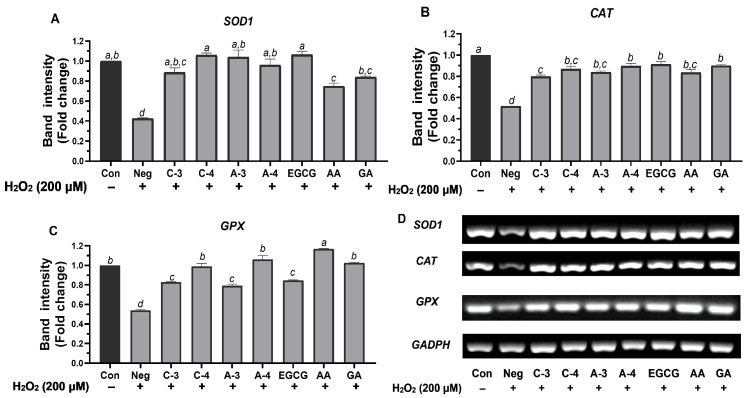
Effects of Brazilian pepper extracts on antioxidant gene expression in hydrogen peroxide (H_2_O_2_)-induced HaCaT cells. (**A**) *SOD1*, (**B**) *CAT*, (**C**) *GPX* mRNA expression levels, and (**D**) representative gel images. HaCaT cells were pre-treated with extracts (50 µg/mL) or standards (epigallocatechin gallate (EGCG), gallic acid (GA), ascorbic acid (AA); 25 µM) prior to exposure to H_2_O_2_ (200 µM). Control = untreated cells; NEG = negative control (cells treated with H_2_O_2_ only). mRNA expression levels were normalized to *GAPDH* as the housekeeping gene. Data are presented as mean ± SD (*n* = 3). Statistical analysis was performed using one-way ANOVA followed by Tukey’s test. Bars with different letters indicate significant differences (*p* < 0.05).

Various samples (C-3, C-4, A-3, A-4) and the reference standard (EGCG, AA, GA) were tested. Gene expression levels were normalized to GAPDH and expressed as fold change relative to the untreated control. Treatment with H_2_O_2_ alone significantly suppressed the expression of all three antioxidant enzymes. The results are presented in Figure 3. The untreated control group consistently showed high expression levels for all three genes. Conversely, H_2_O_2_ treatment alone consistently and significantly suppressed the expression of all three antioxidant enzymes, confirming the induction of oxidative stress at the gene expression level. This is visually represented by the much lower bars for the H_2_O_2_ group compared to the control, and by the reduced band intensity in the gel images.

The expression level of the *SOD1* gene, a critical antioxidant enzyme involved in the dismutation of superoxide radicals, was markedly suppressed by H_2_O_2_ treatment (fold change: 0.43 ± 0.00), confirming the induction of oxidative stress in HaCaT cells (Figure 3A). All tested samples and reference standards significantly restored *SOD1* expression compared to the H_2_O_2_-treated group. Notably, the C-4 extract exhibited the strongest recovery among the plant-derived samples (fold change: 1.06 ± 0.03), showing no significant difference compared to the untreated control and demonstrating a similar level of response to the standard antioxidant EGCG (fold change: 1.06 ± 0.05). A-3 extract also induced a high level of *SOD1* (fold change: 1.04 ± 0.10), which was not different from the control or C-4 extract and EGCG. A-4 demonstrated moderate efficacy (fold change: 0.96 ± 0.08), which was significantly higher than H_2_O_2_ but significantly lower than C-4, A-3, and EGCG. On the other hand, C-3 induced a lower expression level (fold change: 0.89 ± 0.06), which was significantly higher than H_2_O_2_ but lower than most other treatments. Among the standards, AA showed moderate effects (fold change: 0.79 ± 0.06), while GA induced a higher expression level (fold change: 0.92 ± 0.24), with both being significantly different from H_2_O_2_.

The *CAT* gene, which plays a crucial role in detoxifying hydrogen peroxide, was significantly downregulated following H_2_O_2_ exposure, with a fold change of 0.52 ± 0.00 (Figure 3B), indicating oxidative stress-induced suppression. Among the plant extracts tested, the A-4 extract demonstrated the highest induction of *CAT* expression (fold change: 0.90 ± 0.04), showing no significant difference from EGCG (fold change: 0.91 ± 0.04), the reference antioxidant. Another Brazilian pepper extract, C-4, also restored *CAT* expression effectively (fold change: 0.87 ± 0.03), suggesting a strong antioxidant capacity and no significant difference from A-4 and EGCG. A-3 extract and AA showed moderate activity (*CAT* expression of 0.84 ± 0.02 and 0.87 ± 0.04, respectively), with no significant difference among themselves or from A-4, C-4, and EGCG. In contrast, C-3 extract and GA induced lower expression levels (fold change: 0.79 ± 0.02 and 0.80 ± 0.36, respectively). These findings highlight the potential of A-4, C-4, and EGCG as the most effective treatments in restoring *CAT* expression under oxidative stress conditions.

*GPX* gene expression, a key indicator of cellular antioxidant defense, was significantly decreased by H_2_O_2_ treatment (fold change: 0.54 ± 0.01), confirming oxidative stress in HaCaT cells (Figure 3C). Among all tested treatments, AA exhibited the strongest upregulation of *GPX* (fold change: 1.09 ± 0.08), demonstrating efficacy comparable to the untreated control and significantly greater than most other tested treatments. Notably, A-4 extract exhibited the highest level of *GPX* induction among the Brazilian pepper samples (fold change: 1.06 ± 0.06), followed by C-4 extract (fold change: 0.99 ± 0.04) and GA (fold change: 1.03 ± 0.00), all of which supported a notable upregulation of *GPX*. EGCG, while well known for its antioxidant properties, induced *GPX* expression moderately (fold change: 0.85 ± 0.01), which was significantly lower than AA, A-4, C-4, and GA. C-3 and A-3 extracts showed relatively lower expression levels (fold change: 0.83 ± 0.01 and 0.79 ± 0.03, respectively), significantly higher than H_2_O_2_ but lower than the most effective treatments. Based on these observations, the A-4 extract emerges as the most promising plant-based treatment for enhancing *GPX* expression under oxidative stress, with efficacy approaching that of ascorbic acid and surpassing EGCG in this pathway.

In summary, among the Brazilian pepper extracts, C-4 extract showed strong and consistent efficacy across *SOD1* and *CAT* pathways, often performing comparably to the positive control EGCG. A-4 extract demonstrated strong and broad effects. Its efficacy for *CAT* expression was comparable to that of EGCG, AA, and GA, while for *GPX* expression, it was comparable to GA. A-3 extract also showed promising activity, especially for *SOD1*, though it was generally less potent than C-4 and A-4 extracts in other genes. C-3 extract exhibited comparatively weaker activities across all three genes but still provided a significant protective effect compared to H_2_O_2_ alone. These findings suggest that both C-4 and A-4 extracts are promising candidates for antioxidant capacity.

## 3. Discussion

Brazilian pepper (*S. terebinthifolius* Raddi) has gained increasing attention in recent years due to its traditional medicinal uses and emerging evidence of its rich phytochemical composition. In northern Thailand, particularly Chiang Mai province, this plant is locally available and commonly consumed with food, reflecting both its culinary and ethnobotanical significance. This study performed a comparative analysis of the scavenging activity of *S. terebinthifolius* leaf extracts prepared by three distinct methods: CE, ASE, and PEF approaches, using both water and 50% (*v*/*v*) ethanol as solvents. Our findings revealed that CE using 50% (*v*/*v*) ethanol with an extended contact time (120 min), specifically the C-4 extract, consistently yielded the highest extraction yield, TPC, TFC, and exhibited superior antioxidant activities in both ABTS and ORAC assays, alongside robust cellular antioxidant responses. This result is consistent with earlier reports on other medicinal plants, such as *Camellia sinensis* and *Vitis vinifera*, where prolonged maceration with hydroalcoholic solvents enhanced recovery of phenolic compounds and antioxidant potential [35,36]. This suggests that, despite its simplicity and longer duration, the CE method may be more effective for maximizing the recovery of bioactive compounds from Brazilian pepper leaves compared to the more advanced ASE and PEF techniques. This efficiency is largely attributed to the extended contact time between the solvent and plant matrix under mild conditions, which facilitates diffusion-driven mass transfer without thermal degradation of thermolabile phytochemicals [37]. Additionally, the application of magnetic stirring can further improve extraction efficiency by enhancing solvent penetration and plant–solvent interaction [38]. Consistent with this mechanism, pomegranate peel and walnut septum systems have reported higher polyphenol recoveries when dynamic maceration (continuous stirring) was used and/or when stirring speed was increased, reflecting intensified solvent–matrix interactions [39,40,41].

In contrast, PEF did not enhance phenolic extraction compared to CE, despite its proposed advantage of inducing electroporation, which increases membrane permeability and facilitates the release of phenolic compounds [42]. A plausible explanation is that phenolic compounds released during PEF in aqueous systems are highly prone to oxidation and degradation [43], potentially counteracting the benefits of membrane permeabilization. Similar findings have been reported for *Apium graveolens* and *Ruta chalepensis* [44], where ethanol-based CE outperformed PEF in terms of phenolic recovery. These observations suggest that solvent polarity and oxygen exposure strongly influence the efficacy of PEF extraction.

Beyond the extraction method, the solvent system critically determined extraction efficiency across all techniques (CE, ASE, and PEF). The superior performance of 50% (*v*/*v*) ethanol over water can be attributed to its intermediate polarity, which enables efficient solubilization of both hydrophilic and lipophilic phenolics. The dual chemical nature of ethanol, combining a polar hydroxyl group with a non-polar ethyl moiety, facilitates diverse interactions with polyphenolic structures, whereas water alone, with its restricted polarity profile, is less effective [45]. In addition, ethanol improves extraction efficiency by disrupting cellular structures and reducing surface tension, thereby improving tissue wetting and mass transfer [46,47]. Consistently, a previous study reported that the highest content of total phenolic compounds and antioxidant activity from grape stem extracts was obtained using 50% (*v*/*v*) ethanol, compared to water or other ethanol concentrations [48].

Although ASE is designed to improve extraction efficiency by applying high temperatures and pressure, it produced lower yields of TPC and TFC compared to CE with 50% (*v*/*v*) ethanol. This may be explained by the relatively short extraction time employed in ASE in the present study. Similar findings have been reported for grape skins and seeds, where extending the ASE static time yielded higher polyphenol recoveries than short-duration extractions [49]. These observations suggest that extraction time exerts a stronger influence than temperature under the tested conditions. Indeed, extending CE from 15 to 120 min (C-3 vs. C-4 extract) markedly increased TPC, TFC, and antioxidant activity, the critical role of time-dependent diffusion in phenolic recovery. Interestingly, although C-3 extract contained relatively high levels of certain compounds (e.g., gallic acid, caffeic acid, and epicatechin), its overall antioxidant efficacy was weaker than C-4 extract, likely due to synergistic interactions among compounds and the presence of *p*-coumaric acid in C-4, consistent with prior evidence that flavonoid-phenolic acid synergism enhances antioxidant effects [50,51,52].

Our results suggest that extraction time had a stronger effect on the efficiency of compound recovery than temperature, particularly in methods performed at ambient conditions such as CE and PEF. For instance, extending the extraction time from 15 to 120 min significantly improved TPC, TFC, and antioxidant activity in CE (C-3 vs. C-4 extract), despite the constant temperature. Conversely, temperature changes in ASE showed a moderate effect, suggesting that prolonged contact between solvent and plant material plays a more crucial role in maximizing compound diffusion and extraction under mild conditions.

The antioxidant potential of Brazilian pepper extracts, assessed by ABTS and ORAC assays, also varied with extraction technique and solvent. Consistent with our yield and TPC and TFC results, the highest antioxidant activities were obtained from CE using 50% (*v*/*v*) ethanol, especially under extended extraction times (e.g., C-4 extract). The ABTS assay reflects scavenging of the ABTS radical cation, accounting for both hydrophilic and lipophilic antioxidants, while ORAC quantifies peroxyl radical scavenging via hydrogen atom transfer (HAT) mechanisms, providing sensitivity to chain-breaking antioxidants [53]. Strong performance of C-3 and C-4 extracts across both assays suggests that CE effectively recovers a broad spectrum of phenolics acting through multiple antioxidant pathways. Moreover, LC-MS analysis confirmed that C-4 extract contained a diverse profile of bioactive compounds, including gallic acid, caffeic acid, rutin, rosmarinic acid, and epicatechin, all known for potent antioxidant and cytoprotective effects [54,55,56,57,58]. Interestingly, C-3 extract, also obtained by CE but for a shorter time, shared similar compounds with C-4 extract (except for *p*-coumaric acid) and even had higher levels of gallic acid, caffeic acid, and epicatechin. Nevertheless, its overall antioxidant activity and antioxidant gene expression were lower, likely due to insufficient extraction time limiting the total yield of bioactive compounds.

In addition to these in vitro antioxidant assays and phytochemical profiling, the bioefficacy of Brazilian pepper extracts was further validated through gene expression analysis in HaCaT cells under oxidative stress. Treatment with 200 µM H_2_O_2_ successfully induced oxidative stress, reducing cell viability by ~50% and significantly downregulating endogenous antioxidant genes, including *SOD1*, *CAT*, and *GPX*. These molecular changes, coupled with reduced viability, are consistent with established models of H_2_O_2_-induced oxidative damage in keratinocytes [34]. The primary objective was to compare the ability of various Brazilian pepper extracts (C-3, C-4, A-3, A-4) and reference antioxidant standards (EGCG, AA, GA) to counteract this H_2_O_2_-induced gene suppression. For *SOD1*, H_2_O_2_ reduced expression to a 0.43-fold change, while all treatments restored it significantly. C-4 (1.06-fold) and A-3 (1.04-fold) performed comparably to EGCG (1.06-fold), indicating strong efficacy against superoxide radicals. For *CAT*, H_2_O_2_ downregulated expression to 0.52-fold; A-4 (0.90-fold) and C-4 (0.87-fold) restored it to levels statistically similar to EGCG (0.91-fold), while A-3 and AA showed moderate efficacy. For *GPX*, H_2_O_2_ suppressed expression to 0.54-fold, but AA showed the strongest recovery (1.09-fold), followed by A-4 (1.06-fold) and C-4 (0.99-fold). Notably, A-4 exceeded EGCG (0.85-fold), suggesting a specific advantage for certain extracts in GPX-mediated defense.

The significant upregulation of *SOD1*, *CAT*, and *GPX* highlights the potential of Brazilian pepper extracts, particularly C-4 extract, to enhance endogenous antioxidant defenses. These enzymes act synergistically to neutralize ROS and maintain redox balance [59]. *SOD1* acts as the first line of defense, transforming superoxide radicals. *CAT* then efficiently converts hydrogen peroxide, while *GPX* is crucial for detoxifying both hydrogen peroxide and organic peroxides [5]. The synergistic action of these enzymes is vital for cellular integrity. The diverse profile of identified phenolic compounds within C-4 extract likely contributes to its broad-spectrum antioxidant effects through multiple mechanisms. For instance, gallic acid neutralizes free radicals, caffeic acid chelates metal ions and inhibits hydroxyl radical formation, rutin attenuates oxidative stress by inhibiting nuclear factor kappa-light-chain-enhancer of activated B cells’ (NF-κB) activation, rosmarinic acid activates the nuclear factor erythroid 2-related factor 2 (Nrf2) pathway (enhancing *SOD*, *CAT*, and *GPX* expression), and epicatechin neutralizes ROS by donating electrons [54,55,56,57,58]. The similarity of gene activation patterns between C-4 extract and EGCG further supports the concept that polyphenol-rich extracts, efficiently recovered via conventional extraction, can modulate endogenous antioxidant pathways as effectively as established standards. This dual mechanism directs ROS neutralization combined with gene-level modulation, likely explaining the robust antioxidant efficacy of C-4 extract.

Nevertheless, certain limitations should be acknowledged. First, ROS levels were not directly measured, and future studies incorporating assays such as DCFH-DA are warranted. Second, only HaCaT cells were employed, which may not fully represent responses in other skin cell types or in vivo conditions. Third, the extracts contained complex mixtures of phenolics, and the relative contribution of individual compounds remains unclear. Finally, extraction conditions were tested within a narrow range of parameters; broader optimization (e.g., solvent ratios, PEF field strengths, ASE cycles) may reveal different outcomes. Addressing these limitations will be crucial for confirming the mechanistic basis and translational potential of Brazilian pepper extracts as antioxidant agents.

In summary, this study demonstrates that C-4 and A-4 extracts are highly promising candidates for antioxidant-based applications. C-4 extract consistently restored antioxidant gene expression to levels comparable with EGCG, while A-4 extract showed broad and potent effects, particularly for *CAT* and *GPX*. Although C-3 extract was less effective, it still offered significant protection compared with the oxidative control. Future research should aim to isolate the active constituents, clarify their molecular mechanisms, particularly Nrf2-related pathways, and validate these effects in more complex in vivo models.

## 4. Materials and Methods

### 4.1. Chemicals and Reagents

Folin-Ciocalteu’s phenol reagent, ABTS (2,2′-azino-bis(3-ethylbenzothiazoline-6-sulfonic acid)) reagent, quercetin, Trolox, gallic acid, potassium persulfate (K_2_S_2_O_8_), fluorescein, and aluminium chloride (AlCl_3_) were obtained from Sigma-Aldrich (Steinheim, Baden-Württemberg, Germany). Sodium carbonate anhydrous (Na_2_CO_3_) and 2,2′-azobis(2-amidinopropane) dihydrochloride (AAPH) were sourced from Thermo Fisher Scientific Inc. (Thermo Fisher Scientific, Kandel, Germany). EGCG was purchased from Biosynth (Carbosynth, Louisville, KY, USA). Dulbecco’s modified eagle medium (DMEM), fetal bovine serum (FBS), and 1% antibiotics (100 mg/mL streptomycin and 100 U/mL penicillin) were obtained from Gibco Life Technologies (Thermo Fisher Scientific, Waltham, MA, USA).

### 4.2. Sample Preparation

The leaves of the edible plant Brazilian pepper (*S. terebinthifolius* Raddi) were purchased from local markets in Chiang Mai, Thailand (18.3882° N, 98.3840° E) in January 2023. A herbarium voucher specimen of Brazilian pepper (PNPRDU66014) was deposited in the Pharmaceutical and Natural Products Research and Development Unit, Faculty of Pharmacy, Chiang Mai University. The leaves were washed and air-dried in the shade for three days. Afterward, the leaves were dried at 50 °C for 3–5 days in a hot air oven and then ground into a fine powder before extraction using each method.

### 4.3. Extraction Methods

CE, ASE, and PEF extraction methods were used to extract Brazilian pepper. Water and 50% (*v*/*v*) ethanol were used as solvents for each extraction. The other parameters were varied between 15 min and 120 min for time duration, and between 40 °C and 120 °C for the temperature. The code and plant extraction method are in Table 3.

#### 4.3.1. Conventional Extraction

CE was adapted from Martí-Quijal et al. with some modifications [60]. Briefly, 3 g of dried leaf powder (1:66.7 *w*/*v*) was suspended in 200 mL of solvent (water or 50% (*v*/*v*) ethanol) and stirred at 700 rpm using a magnetic stirrer. The extraction time was varied with temperature fixed at 25 °C. The extraction solutions were then filtered using Whatman filter paper No. 1 and stored at 4 °C until further analysis.

#### 4.3.2. Accelerated Solvent Extraction

ASE was adapted from a previous study with some modifications [61]. In brief, 3 g of leaf powder was mixed in a 1:2 ratio with diatomaceous earth (*w*/*w*) using a mortar and transferred into a stainless-steel extraction cell. At 40 °C or 120 °C, extractions were performed with either 50% (*v*/*v*) ethanol or distilled water as solvents, under a constant pressure of 1500 psi using a DIONEX ASE 150 system (Thermo Fisher Scientific Inc., Sunnyvale, CA, USA). The static extraction was set for 5 min per cycle and repeated for three cycles. The extracts were collected in glass vials and kept at 4 °C until further analyses.

#### 4.3.3. Pulsed Electric Fields Extraction

PEF extraction was modified from a previous study [61]. Initially, 6 g of leaf powder was suspended in 200 mL of water and subjected to a specific energy of 100 kJ/kg at an electric field strength of 3 kV/cm. The treatment applied 45 pulses, each with a pulse duration of 100 μs and a frequency of 2 Hz (unipolar square wave pulse), using PEF-Cellcrack III equipment (German Institute for Food Technology (DIL) equipment (ELEA, Quakenbrück, Osnabrück, Germany)) located at the Faculty of Pharmacy, University of Valencia, Valencia, Spain. The distance between electrodes was 10 cm. Conductivity was measured before and after treatment using a ProfiLine Cond 3310 portable conductivity meter (Xylem Analytics Germany Sales GmbH & Co. KG, Weilheim, Germany). After PEF treatment, an additional 200 mL of solvent (water or 50% (*v*/*v*) ethanol) was added to adjust the final solid-to-solvent ratio to 1:66.7 (*w*/*v*), and the suspension was stirred with a magnetic stirrer at 700 rpm for the designated extraction times. The extracts were then filtered through Whatman filter paper No. 1 and stored at 4 °C until further analysis.

### 4.4. Phytochemical Analysis

#### 4.4.1. Total Phenolic Content

The total phenolic content was determined using the colorimetric Folin-Ciocalteu technique [61]. Briefly, 100 μL of sample solutions was mixed with 100 μL 50% Folin-Ciocalteu reagent and 3000 μL of 4% Na_2_CO_3_ in a cuvette. The mixture was then incubated in the dark for 1 h. After the reaction period, the absorbance was measured at 765 nm using a Perkin-Elmer UV/VIS Lambda 2 spectrophotometer (Perkin-Elmer, Jügesheim, Germany). A calibration curve was constructed using gallic acid as the standard. The total phenolic content was expressed as mg GAE/g DM.

#### 4.4.2. Total Flavonoid Content

The aluminium chloride colorimetric assay was used to determine the total flavonoid content [62]. Briefly, 100 μL of the sample solution was mixed with 100 μL of 2% aluminium chloride solution. The mixture was then incubated for 30 min at room temperature. After incubation, the absorbance was measured at 415 nm using a FLUOstar OMEGA microplate reader (BMG Labtech GmbH, Ortenberg, Germany). A calibration curve was constructed using quercetin as the standard. The results were expressed as mg QE/100 g DM.

#### 4.4.3. Identification of Constituents by LC-MS Analysis

The characterization and quantification of polyphenols in the extracts were performed using LC-MS, following a previously established protocol [63]. Briefly, an LC-MS Agilent 1260 Infinity II series, coupled with an electrospray ion (ESI) quadrupole mass spectrometry 6130 (Agilent Tech., Santa Clara, CA, USA), was used. The LC system was equipped with a binary pump, autosampler, and Ultra C18 column (5 μm 4.6 × 250 mm, Restek, Bellefonte, PA, USA). The mobile phase consisted of two solvents, A (0.2% acetic acid in 5% MeOH) and B (0.2% acetic acid in 50% acetonitrile). The flow rate was 0.5 mL/min with an injection volume of 20 µL of the sample. The gradient program was as follows: 0–45 min, 10–20% solvent B; 45–85 min, 20–55% solvent B; 85–97 min, 55–100% solvent B; 97–110 min, 100% B; followed by a re-equilibration to the initial condition over 10 min, and the mass spectrometer operated in negative selected ion monitoring (SIM) mode. The mass spectrometry settings included a nitrogen flow rate of 12 L/min, a drying gas temperature of 350 °C, a nebulizer pressure of 60 psi, a capillary voltage of 3000 V, a fragmentor voltage of 70 V, and full scan spectra from 100 to 1200 *m*/*z* with 250 ms/spectrum.

### 4.5. Antioxidant Capacity

#### 4.5.1. ABTS Radical Scavenging Activity

ABTS radical scavenging was used to determine total antioxidant capacity (TEAC) as described [64]. To prepare the ABTS reagent, 140 mM potassium persulphate (440 μL) was added to 7 mM ABTS solution (25 mL) and kept in darkness for 12–16 h to generate free radicals. Then, the ABTS reagent was diluted with distilled water (1:100) until the absorbance at 734 nm was about 0.70 ± 0.02 at 30 °C. The sample solution or trolox (100 μL) was placed into the cuvette with ABTS reagents (2500 μL). After 10 min in the darkness, the color of the reaction was measured at 734 nm. The results were calculated as µmol TE/g DM.

#### 4.5.2. Oxygen Radical Absorbance Capacity (ORAC) Assay

The ORAC antioxidant capacity was measured based on the hydrogen atom transfer mechanism using fluorescein as the fluorescent probe [65]. Briefly, the sample solution or standard solution (50 μL) (Trolox) was placed into the 96-well plate with fluorescein (50 μL) for 10 min at 37 °C. Then, the reaction was initiated by 2,2′-azobis(2-amidinopropane) dihydrochloride (AAPH) (25 μL). The fluorescence measurements (excitation wavelength 485 nm and emission wavelength 520 nm) were taken every 60 s for 120 min. The results were calculated as µM TE/100 g DM.

### 4.6. Cell Cultures and Assessment of Cell Viability

#### 4.6.1. Cell Culture

HaCaT cells were cultured in DMEM GlutaMAX^TM^ containing 10% FBS and 1% antibiotics (100 mg/mL streptomycin and 100 U/mL penicillin). The HaCaT cell line was kindly obtained from Assoc. Prof. Dr. Chuda Chittasupho, Faculty of Pharmacy, Chiang Mai University. Cells were maintained at 37 °C in a humidified incubator containing 5% CO_2._ The culture medium was replaced every 2–3 days until the cells achieved 80% confluence. For all subsequent experiments, only healthy and actively growing HaCaT cells with an estimated viability greater than 80% were used. Viability was routinely monitored by microscopic examination of cell morphology to ensure that cultures were free from detached or morphologically abnormal cells prior to treatment. In addition, an untreated control group was included in every experiment to serve as the baseline for comparison.

#### 4.6.2. Cytotoxic Effect of Brazilian Pepper Extracts

The extracts were investigated for cell viability of HaCaT cells using the SRB assay, slightly modified from the method as described previously [66]. Briefly, cells were seeded in 96-well plates for 24 h at a density of 20,000 cells/well. Then, the medium was removed and replaced with either an extract solution (5–1000 µg/mL) or the reference standard (0–200 µM) for 24 h. Cells were fixed with 50 μL of 50% trichloroacetic acid (TCA) at 4 °C for 1 h, washed with water, and air-dried. The cells were stained with 0.04% SRB solution for 30 min, followed by four washes with 1% acetic acid to remove unbound dye. The protein-bound dye was solubilized using 10 mM Tris base, and absorbance was measured at 515 nm using a SPECTROstar Nano microplate reader (BMG Labtech, Ortenberg, Germany). The percentage of cell viability was calculated using the following formula (A_515_ of treated cells/A_515_ of control cells) × 100%. In accordance with ISO 10993-5 [67], cell viability values above 80% were considered non-cytotoxic [68].

#### 4.6.3. Effect of H_2_O_2_ on Cell Viability of HaCaT Cells

To identify the H_2_O_2_ concentration that results in an approximately 50% reduction in HaCaT cells viability, a method was employed with slight modifications from Warinhomhoun et al. [34]. Briefly, cells were seeded in 96-well plates for 24 h at a density of 20,000 cells/well, the medium was then replaced with fresh medium containing various concentrations of H_2_O_2_ (0, 50, 100, 200, 400, 800, 1000 μM) and incubated for 24 h. The serum-free medium without H_2_O_2_ served as a control. HaCaT cells were washed twice with PBS after incubation, and viability was subsequently determined using the SRB assay. The H_2_O_2_ concentration that reduced cell viability to approximately 50% was then selected for further analysis [34].

#### 4.6.4. Effect of Brazilian Pepper Extracts on Cell Viability of HaCaT Cells Under Oxidative Stress

To evaluate the protective effect of the extracts against H_2_O_2_-induced cell death, a method was employed with slight modifications from Liu et al. [69]. Following this, cells were pre-treated with the extracts for 2.5 h and then washed with PBS. Subsequently, the cells were exposed to 200 μM H_2_O_2_ (a concentration determined to cause approximately 50% reduction in HaCaT cell viability) and incubated for an additional 24 h. Cells treated with medium were used as the control group. Following incubation, the SRB assay was performed.

### 4.7. RNA Extraction and Semi-Quantitative Reverse Transcriptase Polymerase Chain Reaction

HaCaT cells were pre-treated with extracts at 50 µg/mL, a concentration that maintained 80% cell viability for 2.5 h, and then exposed to H_2_O_2_ (200 μM) for 24 h. Total RNA was extracted using the E.Z.N.A. Total RNA Kit I (Omega Bio-Tek, Norcross, GA, USA) following the manufacturer’s instructions. RNA samples were stored at −80 °C until further use. First-strand cDNA was synthesized from 1 μg of RNA using the MyTaq™ One-Step RT-PCR Kit (Bioline Meridian Bioscience, Eveleigh, NSW, Australia) according to the manufacturer’s protocol. Semi-quantitative RT-PCR was performed using gene-specific primers (Table 4). The amplification conditions were as follows: denaturation at 95 °C for 10 s, annealing (temperature specific for each gene) for 10 s, and extension at 72 °C for 30 s, over 40 cycles. Amplification products were visualized by electrophoresis on a 1.5% agarose gel using the Gel Doc™ EZ System (Bio-Rad Laboratories, Inc., Hercules, CA, USA), and band intensities were analyzed with Image Lab software version 6.1.0 build 7 (Bio-Rad Laboratories, Inc., Hercules, CA, USA).

### 4.8. Statistical Analysis

The results were expressed as the mean ± standard deviation (SD). Statistical analysis was performed using SPSS 17.0 software (SPSS Inc., Chicago, IL, USA) with a one-way ANOVA followed by Tukey’s post hoc test. A *p*-value of less than 0.05 was considered statistically significant.

## 5. Conclusions

This comparative study successfully identified CE with 50% (*v*/*v*) ethanol, particularly the C-4 condition, as the optimal method for *S. terebinthifolius* leaf extracts. The C-4 extraction achieved the highest extraction yield together with the highest phenolic and flavonoid recovery. Moreover, C4 extract showed the strongest antioxidant activity in both ABTS and ORAC assays. Additionally, this extract provided notable antioxidant protection against H_2_O_2_-induced oxidative damage in human keratinocytes by upregulating *SOD1*, *CAT*, and *GPX* levels comparable to standard antioxidants. Our findings highlighted the potential of optimized conventional extraction to produce *S. terebinthifolius* extracts as effective candidates for cosmeceutical applications against oxidative-stress-related skin conditions. Future studies should focus on isolating active compounds, clarifying molecular mechanisms, evaluating stability, and in vivo efficacy to support clinical translation.

## Figures and Tables

**Table 1:** Extraction yield, total phenolic content (TPC), total flavonoid content (TFC), and antioxidant activities (ABTS and ORAC assays) of extracts obtained from different extraction methods and conditions.

Code	Extraction Method	Solvent	(°C)	Time (min)	Yield (%)	TPC (mg GAE/g DM)	TFC (mg QE/100 g DM)	ABTS (µmol TE/g DM)	ORAC (µM TE/100 g DM)
C-1	CE	Water	25	15	16.2 ± 0.18 ^f^	188.45 ± 4.96 ^d^	180.97 ± 6.34 ^e,f^	2353.24 ± 112.51 ^f^	2924.75 ± 57.82 ^d^
C-2			25	120	17.8 ± 0.44 ^e^	246.13 ± 12.31 ^c^	409.01 ± 10.23 ^c,d^	2983.94 ± 50.71 ^d^	2873.19 ± 59.98 ^d,e^
C-3		50% EtOH	25	15	27.4 ± 0.56 ^b^	542.64 ± 27.14 ^b^	2074.69 ± 134.12 ^b^	11,566.38 ± 44.25 ^b^	6358.00 ± 162.74 ^b^
C-4			25	120	29.7 ± 0.48 ^a^	668.56 ± 11.52 ^a^	2629.92 ± 112.61 ^a^	12,645.50 ± 60.31 ^a^	7180.27 ± 101.79 ^a^
A-1	ASE	Water	40	25	13.5 ± 0.12 ^h^	52.11 ± 3.50 ^f^	61.17 ± 2.05 ^f^	639.67 ± 12.31 ^i^	2423.08 ± 175.37 ^f^
A-2			120	25	14.7 ± 0.24 ^g,h^	106.42 ± 4.40 ^e^	282.37 ± 6.70 ^d,e^	2489.02 ± 6.64 ^f^	4890.34 ± 330.55 ^c^
A-3		50% EtOH	40	25	24.1 ± 0.60 ^d^	132.09 ± 5.54 ^e^	474.43 ± 12.38 ^c^	2699.54 ± 12.06 ^e^	6758.86 ± 130.26 ^a,b^
A-4			120	25	25.6 ± 0.28 ^c^	166.01 ± 1.83 ^d^	543.12 ± 6.13 ^c^	3613.60 ± 18.09 ^c^	6770.08 ± 43.73 ^a,b^
P-1	PEF	Water	25	15	8.3 ± 0.80 ^k^	18.67 ± 0.61 ^g^	ND	1151.60 ± 6.36 ^h^	642.34 ± 47.76 ^g^
P-2			25	120	10.0 ± 0.49 ^j^	8.81 ± 0.11 ^g^	ND	1062.28 ± 51.60 ^h^	927.87 ± 6.46 ^g^
P-3		50% EtOH	25	15	11.7 ± 0.14 ^i^	125.09 ± 2.81 ^e^	131.39 ± 3.06 ^e,f^	1616.93 ± 53.48 ^g^	2489.13 ± 211.48 ^e,f^
P-4			25	120	15.0 ± 0.49 ^f,g^	58.47 ± 3.72 ^f^	141.31 ± 2.59 ^e,f^	1543.30 ± 32.80 ^g^	2717.73 ± 26.60 ^d,e,f^

Values are expressed as the mean ± SD; ND is not detected; GAE: gallic acid equivalent; QE: quercetin equivalent; TE: Trolox equivalent. Different letters represent statistical differences according to the Tukey’s HSD method. A value of *p* < 0.05 was considered statistically significant for comparisons among all extracts.

**Table 2:** LC-MS data of polyphenols found in *Schinus terebinthifolius* Raddi leaf extracts.

No.	Compound	MS	Concentrations mg/g Extract			
			C-3	C-4	A-3	A-4
1	Gallic acid	170.20	1.618 ± 0.032	1.300 ± 0.018	1.155 ± 0.005	1.542 ± 0.127
2	*p*-Coumaric acid	164.16	ND	0.588 ± 0.008	0.139 ± 0.020	0.167± 0.007
3	Rutin	610.52	0.031 ± 0.001	0.027 ± 0.002	0.041 ± 0.000	0.011± 0.001
4	Rosmarinic acid	360.31	0.254 ± 0.008	0.254 ± 0.001	0.370 ± 0.002	0.245 ± 0.002
5	Caffeic acid	180.16	0.309 ± 0.118	0.144 ± 0.033	ND	0.193 ± 0.006
6	Epicatechin	290.28	0.073 ± 0.004	0.080 ± 0.002	ND	ND

Values are expressed as mean ± SD; ND indicates not detected; C-3: conventional extraction at 25 °C with 50% (*v*/*v*) ethanol for 15 min; C-4: conventional extraction at 25 °C with 50% (*v*/*v*) ethanol for 120 min; A-3: accelerated solvent extraction at 40 °C with 50% (*v*/*v*) ethanol for 25 min; A-4: accelerated solvent extraction at 120 °C with 50% *(v/v)* ethanol for 25 min.

**Table 3:** Extraction methods and conditions.

Code	Extraction Method	Solvent	Temperature (°C)	Time (min)
C-1	CE	Water	25	15
C-2		Water	25	120
C-3		50% (*v*/*v*) Ethanol	25	15
C-4		50% (*v*/*v*) Ethanol	25	120
A-1	ASE	Water	40	25
A-2		Water	120	25
A-3		50% (*v*/*v*) Ethanol	40	25
A-4		50% (*v*/*v*) Ethanol	120	25
P-1	PEF	Water	25	15
P-2		Water	25	120
P-3		50% (*v*/*v*) Ethanol	25	15
P-4		50% (*v*/*v*) Ethanol	25	120

CE: conventional extraction; ASE: accelerated solvent extraction; PEF: pulsed electric field extraction.

**Table 4:** Sequences of forward and reverse primers used for semi-quantitative RT-PCR analysis.

Primers	Forward Primer	Reverse Primer
*SOD1*	AGGGCATCATCAATTTCGAG	ACATTGCCCAAGTCTCCAAC
*CAT*	CATCGCCACATGAATGGATA	CCAACTGGGATGAGAGGGTA
*GPX*	TTCCCGTGCAACCAGTTTG	GGACGTACTTGAGGGAATTCAGA
*GADPH*	GGAAGGTGAAGGTCGGAGTC	CTCAGCCTTGACGGTGCCATG

## Data Availability

The data presented in this study are available on request from the corresponding author.

## Supplementary Materials

The following supporting information can be downloaded at: https://www.mdpi.com/article/10.3390/molecules30173589/s1, Figure S1: Total ion chromatogram (TIC) of A-3 extract analyzed by LC-MS in negative mode ESI, showing the main peaks of detected compounds; Figure S2: Mass spectral analysis of peak at retention time 4.215 min from A-3 extract; Figure S3: Mass spectral analysis of peak at retention time 4.596 min from A-3 extract; Figure S4: Mass spectral analysis of peak at retention time 5.707 min from A-3 extract; Figure S5: Mass spectral analysis of peak at retention time 72.737 min from A-3 extract; Figure S6: Total ion chromatogram (TIC) of A-4 extract analyzed by LC-MS in negative mode ESI, showing the main peaks of detected compounds; Figure S7: Mass spectral analysis of peak at retention time 4.207 min from A-4 extract; Figure S8: Mass spectral analysis of peak at retention time 4.548 min from A-4 extract; Figure S9: Mass spectral analysis of peak at retention time 5.569 min from A-4 extract; Figure S10: Mass spectral analysis of peak at retention time 7.867 min from A-4 extract; Figure S11: Mass spectral analysis of peak at retention time 73.049 min from A-4 extract; Figure S12: Total ion chromatogram (TIC) of C-3 extract analyzed by LC-MS in negative mode ESI, showing the main peaks of detected compounds; Figure S13: Mass spectral analysis of peak at retention time 4.109 min from C-3 extract; Figure S14: Mass spectral analysis of peak at retention time 4.491 min from C-3 extract; Figure S15: Mass spectral analysis of peak at retention time 5.546 min from C-3 extract; Figure S16: Mass spectral analysis of peak at retention time 5.772 min from C-3 extract; Figure S17: Mass spectral analysis of peak at retention time 7.946 min from C-3 extract; Figure S18: Mass spectral analysis of peak at retention time 9.943 min from C-3 extract; Figure S19: Mass spectral analysis of peak at retention time 73.130 min from C-3 extract; Figure S20: Mass spectral analysis of peak at retention time 97.113 min from C-3 extract; Figure S21: Total ion chromatogram (TIC) of C-4 extract analyzed by LC-MS in negative mode ESI, showing the main peaks of detected compounds; Figure S22: Mass spectral analysis of peak at retention time 4.200 min from C-4 extract; Figure S23: Mass spectral analysis of peak at retention time 4.489 min from C-4 extract; Figure S24: Mass spectral analysis of peak at retention time 5.545 min from C-4 extract; Figure S25: Mass spectral analysis of peak at retention time 5.828 min from C-4 extract; Figure S26: Mass spectral analysis of peak at retention time 8.006 min from C-4 extract; Figure S27: Mass spectral analysis of peak at retention time 10.038 min from C-4 extract; Figure S28. Mass spectral analysis of peak at retention time 73.085 min from C-4 extract.
