# Supplementary material for: Comparative Study on Antioxidant Potential of Schinus terebinthifolius Extracts Prepared by Conventional Extraction, Accelerated Solvent Extraction, and Pulsed Electric Field Method
Source: Molecules. 2025 Sep 2;30(17):3589. doi: 10.3390/molecules30173589 (PMC12430624; doi:10.3390/molecules30173589)
Supplement: Supplementary file 1 [file molecules-30-03589-s001.zip › molecules-3791067-supplementary.pdf]

Article

Comparative Study on Antioxidant Potential of *Schinus terebinthifolius* Extracts Prepared by Conventional Extraction, Accelerated Solvent Extraction, and Pulsed Electric Field Method

Supplementary Information

General Experimental Procedures

The characterization and quantification of polyphenols in the extracts were performed using liquid chromatography-mass spectrometry (LC-MS), following a previously established protocol. Briefly, an LC-MS Agilent 1260 Infinity II series, coupled with an electrospray ion (ESI) quadrupole mass spectrometry 6130 (Agilent Tech., Santa Clara, CA, USA) was used. The LC system was equipped with a binary pump, autosampler, and Ultra C18 column (5 µm 4.6× 250 mm, Restek, Bellefonte, PA, USA). The mobile phase consisted of two solvents, A (0.2% acetic acid in 5% MeOH) and B (0.2% acetic acid in 50% acetonitrile). The flow rate was 0.5 mL/min with an injection volume of 20 µL of the sample. The gradient program was as follows: 0-45 min, 10-20% solvent B; 45-85 min, 20-55% solvent B; 85-97 min, 55-100% solvent B; 97-110 min, 100% B; followed by a re-equilibration to the initial condition over 10 min, and the mass spectrometer operated in negative selected ion monitoring (SIM) mode. The mass spectrometry settings included a nitrogen flow rate of 12 L/min, a drying gas temperature of 350 °C, a nebulizer pressure of 60 psi, a capillary voltage of 3000 V, a fragmentor voltage of 70 V, and full scan spectra from 100 to 1200 m/z with 250 ms/spectrum.

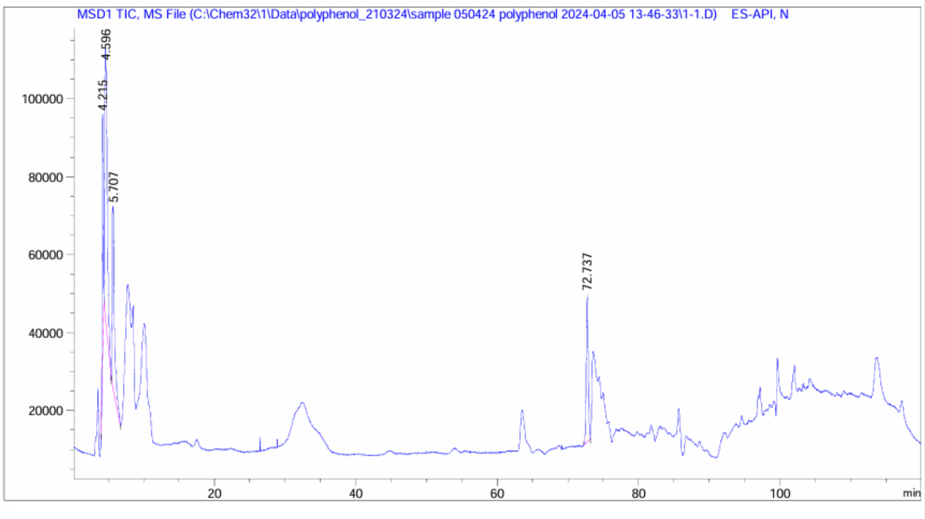

Figure S1. Total Ion Chromatogram (TIC) of A-3 extract analyzed by LC-MS in negative mode ESI, showing the main peaks of detected compounds.

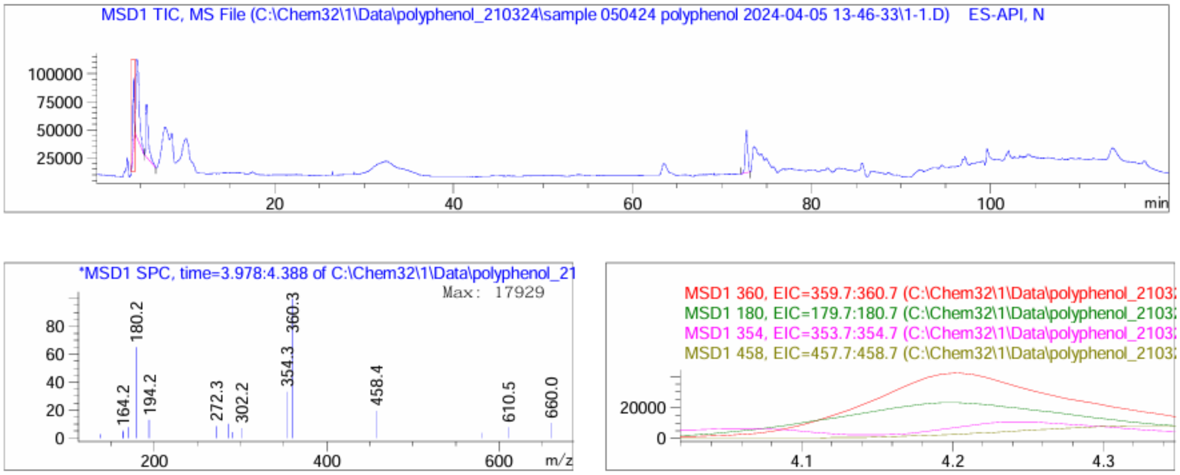


```
Peak #1 at  4.215 min ( 3.978 to  4.389 min)
-> The analysis found 3 components, indicating an impure peak. <-

Component 1:  Peak at Scan 400.8. Top ions are  360 180
Component 2:  Peak at Scan 405.1. Top ions are  354
Component 3:  Peak at Scan 414.5. Top ions are  458
```

Figure S2. Mass spectral analysis of peak at retention time 4.215 min from A-3 extract.

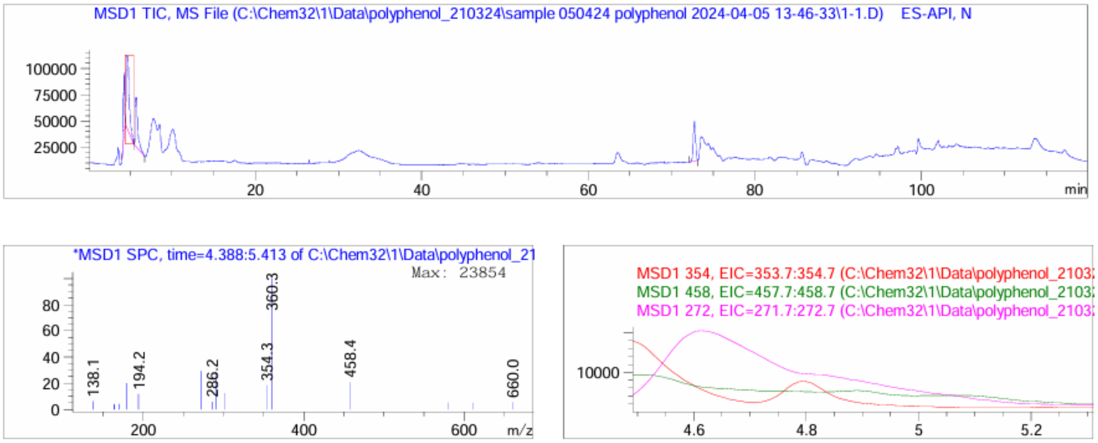


```
Peak #2 at  4.596 min ( 4.389 to  5.413 min)
-> The analysis found 7 components, indicating an impure peak. <-

Component 1:  Peak at Scan 429.0. Top ions are  354
Component 2:  Peak at Scan 430.7. Top ions are  458
Component 3:  Peak at Scan 441.2. Top ions are  272
Component 4:  Peak at Scan 442.8. Top ions are  290
Component 5:  Peak at Scan 449.2. Top ions are  360
Component 6:  Peak at Scan 454.8. Top ions are  302
Component 7:  Peak at Scan 458.2. Top ions are  180
```

Figure S3. Mass spectral analysis of peak at retention time 4.596 min from A-3 extract.

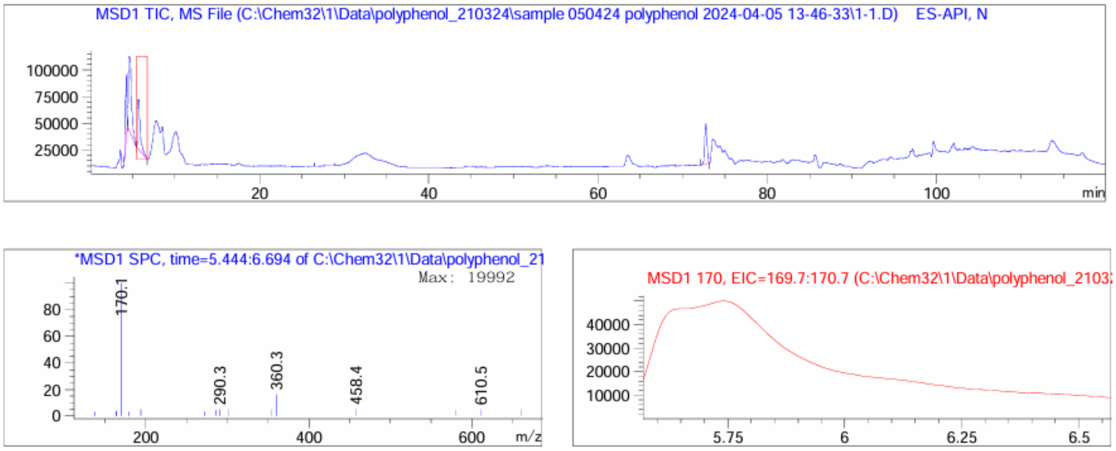


```
Peak #3 at  5.707 min ( 5.444 to  6.694 min)
-> The analysis found only one component, indicating a pure peak. <-
```

Figure S4. Mass spectral analysis of peak at retention time 5.707 min from A-3 extract.

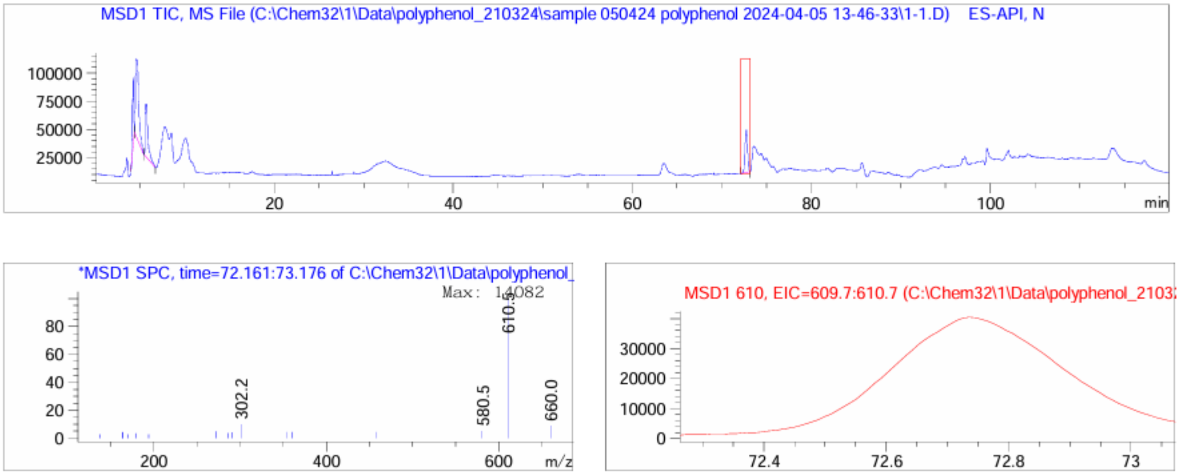


```
Peak #4 at 72.737 min (72.161 to 73.174 min)
-> The analysis found only one component, indicating a pure peak. <-

Component 1:  Peak at Scan 7087.3. Top ions are  610
```

Figure S5. Mass spectral analysis of peak at retention time 72.737 min from A-3 extract.

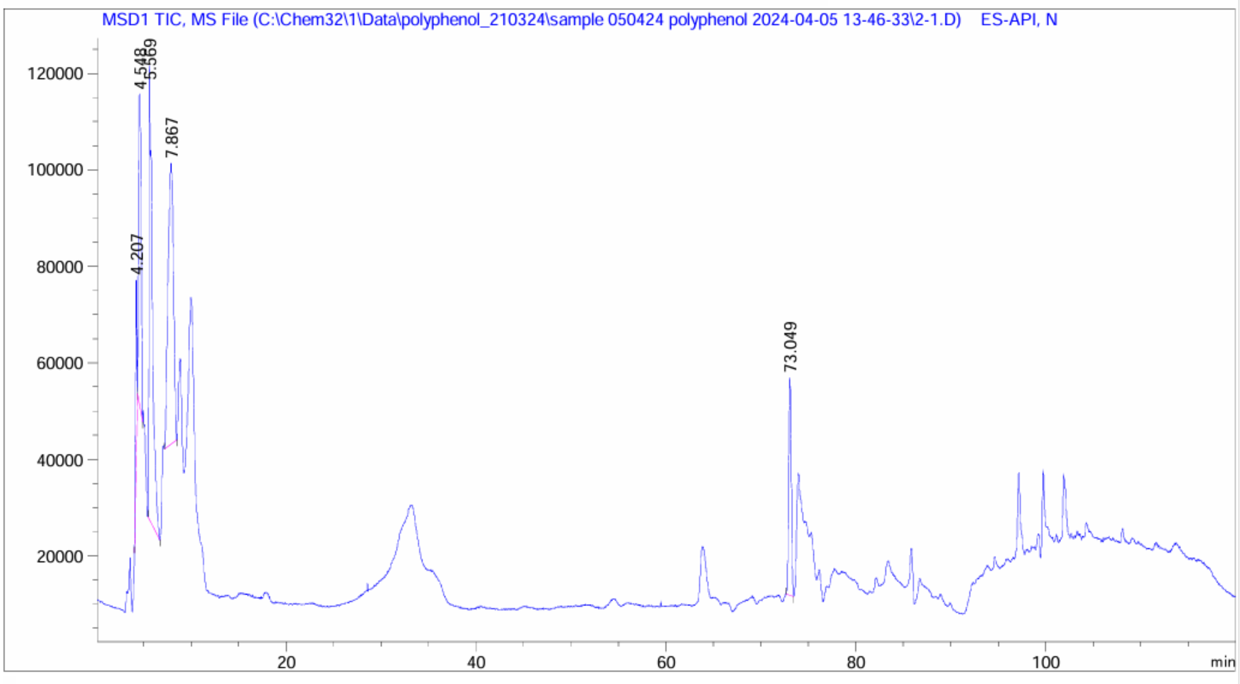

Figure S6. Total Ion Chromatogram (TIC) of A-4 extract analyzed by LC-MS in negative mode ESI, showing the main peaks of detected compounds.

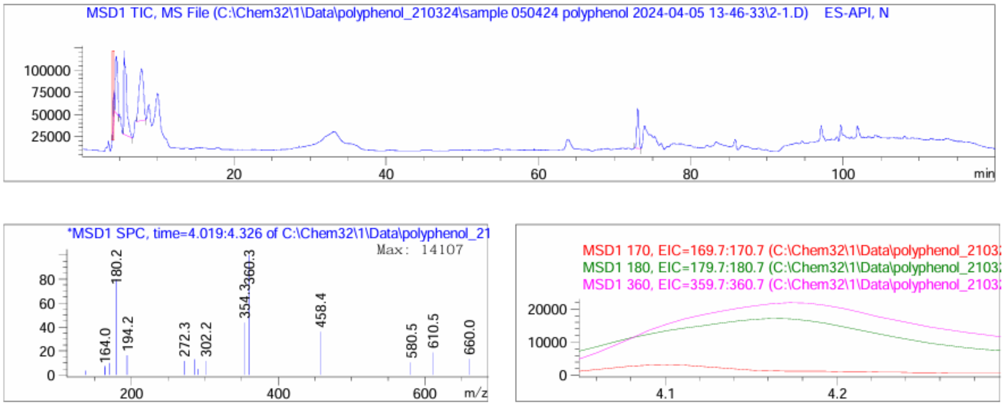


```
Peak #1 at  4.207 min ( 4.019 to  4.326 min)
-> The analysis found 8 components, indicating an impure peak. <-

Component 1:  Peak at Scan 390.8. Top ions are  170
Component 2:  Peak at Scan 397.2. Top ions are  180
Component 3:  Peak at Scan 398.1. Top ions are  360
Component 4:  Peak at Scan 400.1. Top ions are  194
Component 5:  Peak at Scan 401.2. Top ions are  286
Component 6:  Peak at Scan 401.9. Top ions are  354
Component 7:  Peak at Scan 403.8. Top ions are  164
Component 8:  Peak at Scan 405.4. Top ions are  610
```

Figure S7. Mass spectral analysis of peak at retention time 4.207 min from A-4 extract.

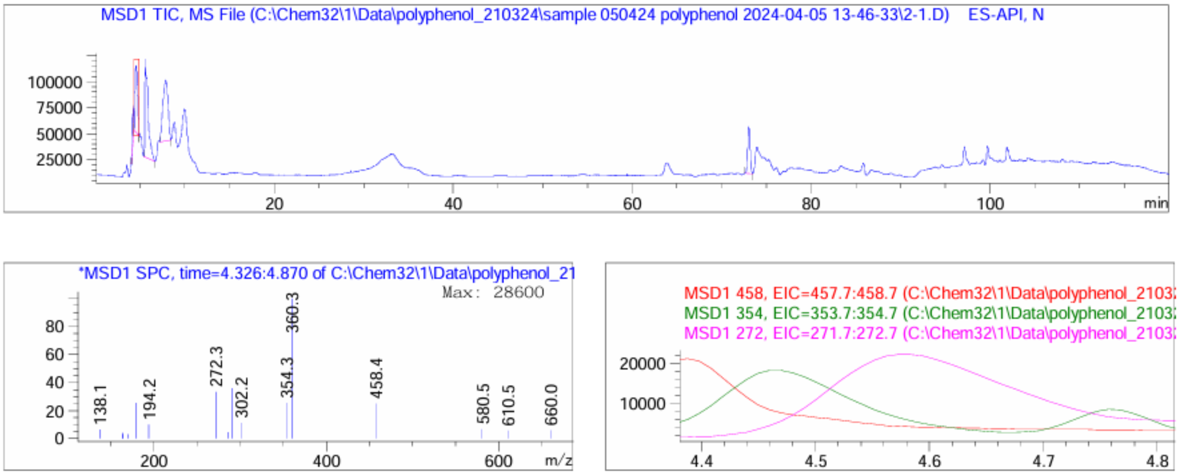


```
Peak #2 at  4.548 min ( 4.326 to  4.870 min)
-> The analysis found 6 components, indicating an impure peak. <-

Component 1:  Peak at Scan 418.9. Top ions are  458
Component 2:  Peak at Scan 426.5. Top ions are  354
Component 3:  Peak at Scan 437.5. Top ions are  272
Component 4:  Peak at Scan 438.9. Top ions are  290
Component 5:  Peak at Scan 444.3. Top ions are  360
Component 6:  Peak at Scan 455.3. Top ions are  180
```

Figure S8. Mass spectral analysis of peak at retention time 4.548 min from A-4 extract.

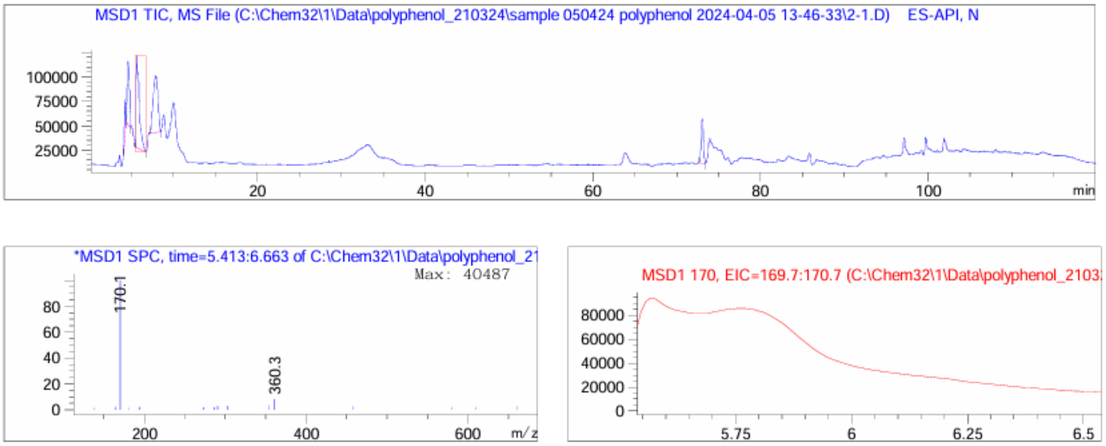


```
Peak #3 at  5.569 min ( 5.413 to  6.663 min)
-> The analysis found only one component, indicating a pure peak. <-

Component 1:  Peak at Scan 534.3. Top ions are  170
```

Figure S9. Mass spectral analysis of peak at retention time 5.569 min from A-4 extract.

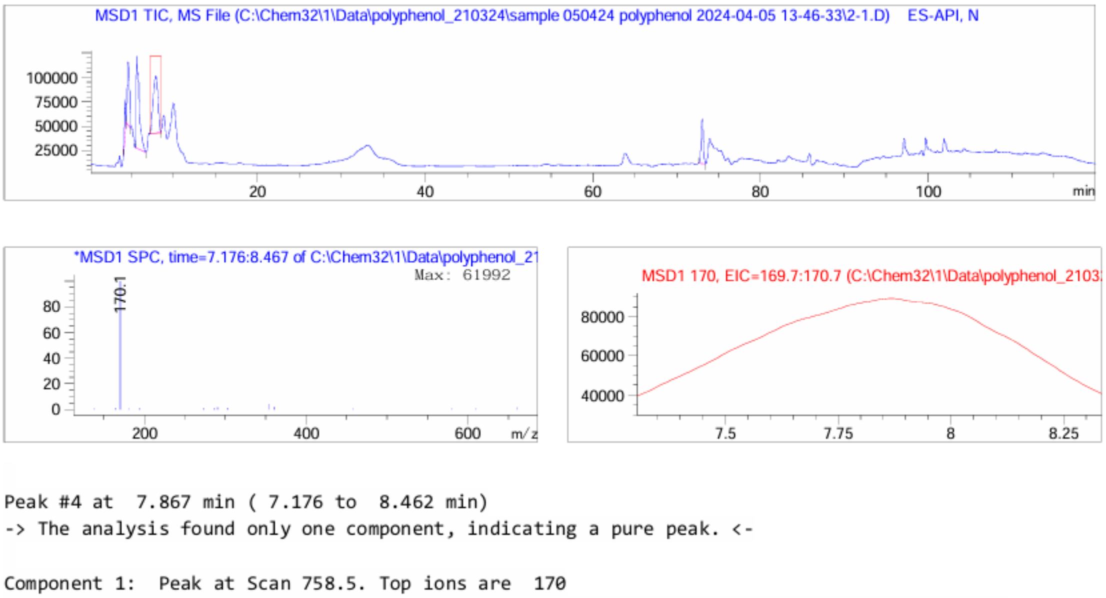

Figure S10. Mass spectral analysis of peak at retention time 7.867 min from A-4 extract.

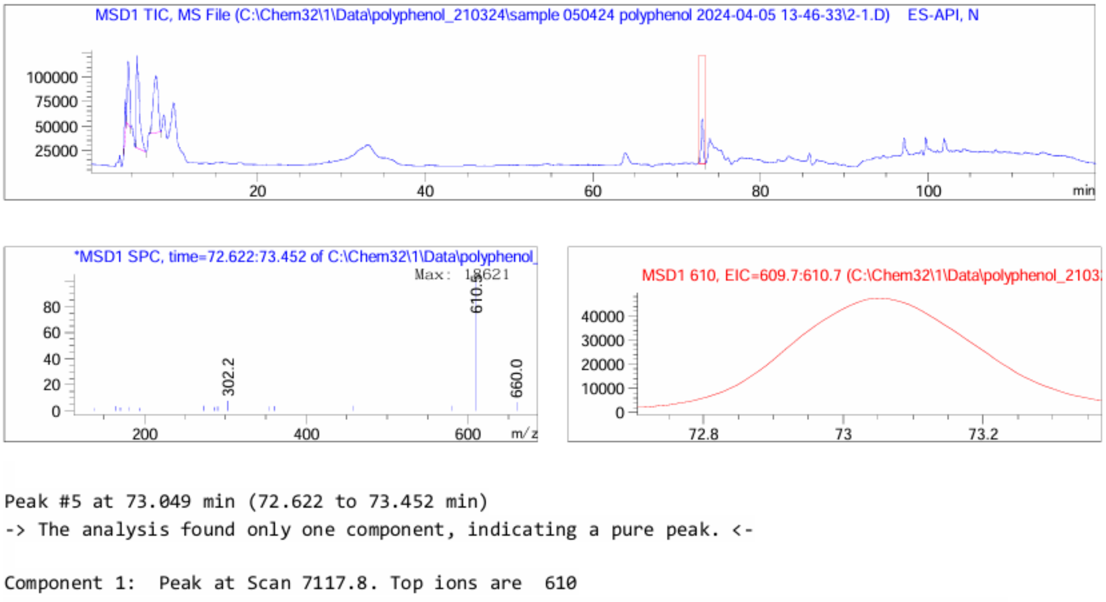

Figure S11. Mass spectral analysis of peak at retention time 73.049 min from A-4 extract.

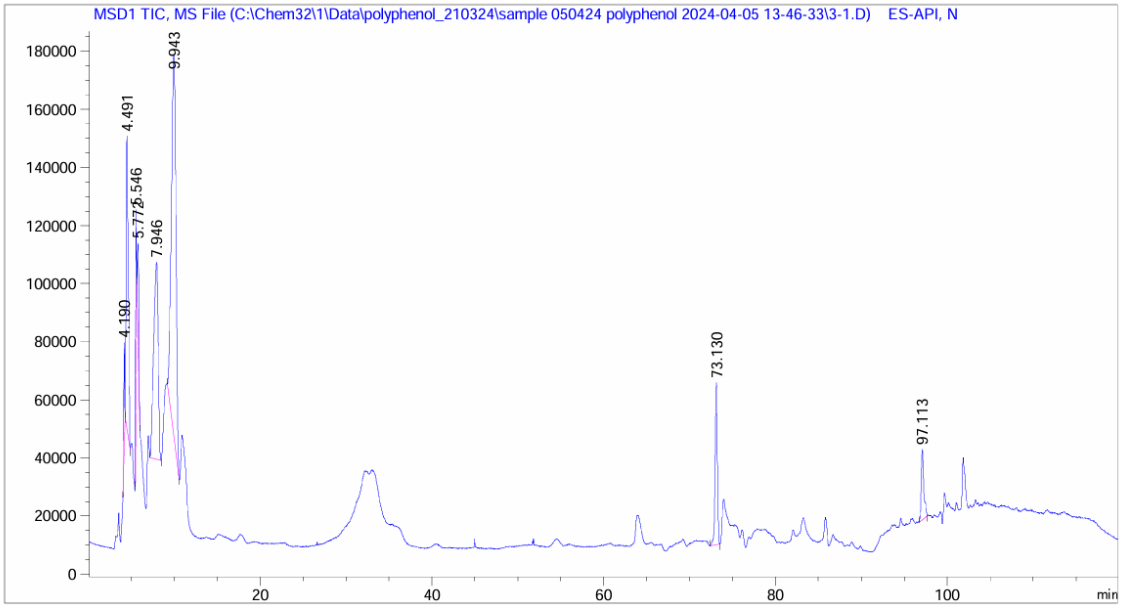

Figure S12. Total Ion Chromatogram (TIC) of C-3 extract analyzed by LC-MS in negative mode ESI, showing the main peaks of detected compounds.

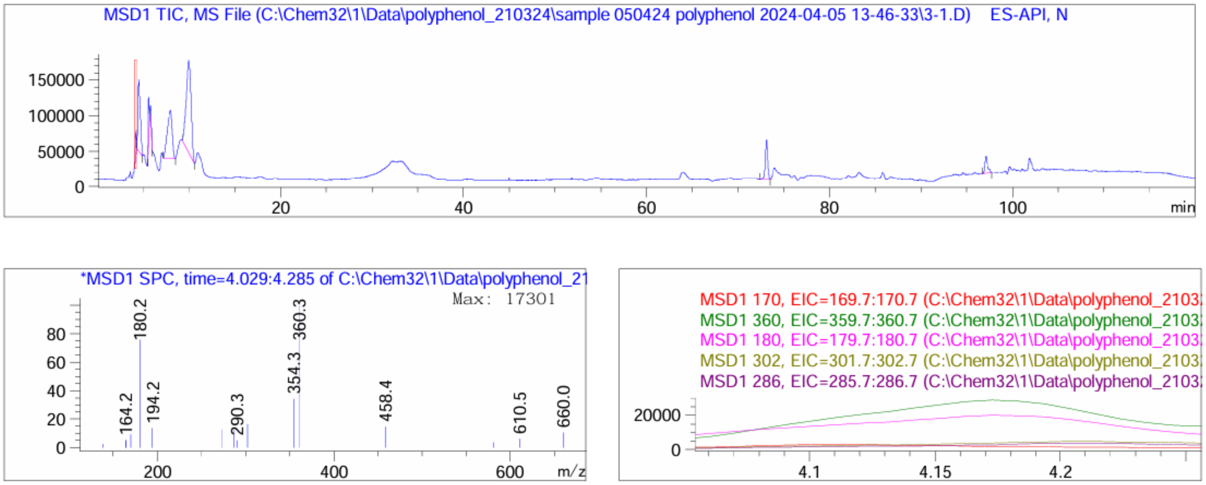


```
Peak #1 at  4.190 min ( 4.029 to  4.281 min)
-> The analysis found 4 components, indicating an impure peak. <-

Component 1:  Peak at Scan 392.0. Top ions are  170
Component 2:  Peak at Scan 398.4. Top ions are  360 180 194
Component 3:  Peak at Scan 401.7. Top ions are  302 286
Component 4:  Peak at Scan 402.4. Top ions are  354
```

Figure S13. Mass spectral analysis of peak at retention time 4.109 min from C-3 extract.

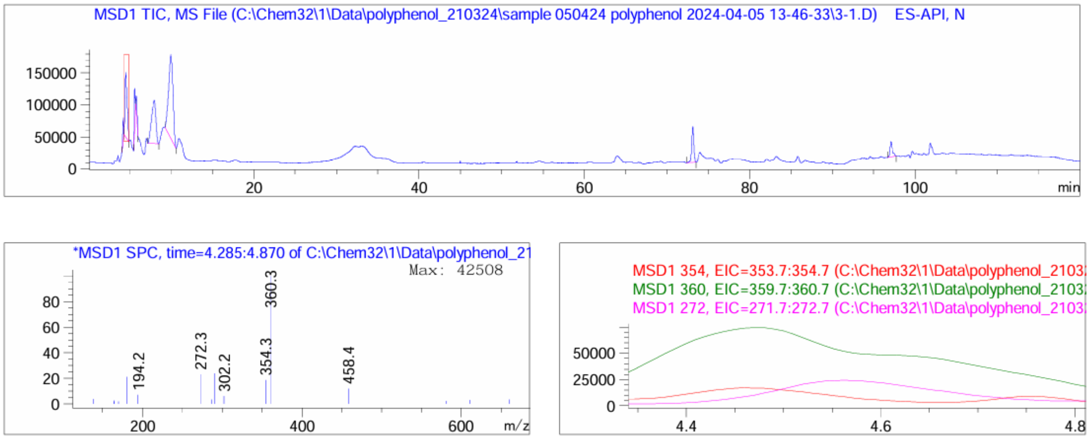


```
Peak #2 at  4.491 min ( 4.281 to  4.870 min)
-> The analysis found 5 components, indicating an impure peak. <-

Component 1:  Peak at Scan 426.4. Top ions are  354
Component 2:  Peak at Scan 427.2. Top ions are  360
Component 3:  Peak at Scan 435.9. Top ions are  272
Component 4:  Peak at Scan 436.8. Top ions are  290
Component 5:  Peak at Scan 455.2. Top ions are  180
```

Figure S14. Mass spectral analysis of peak at retention time 4.491 min from C-3 extract.

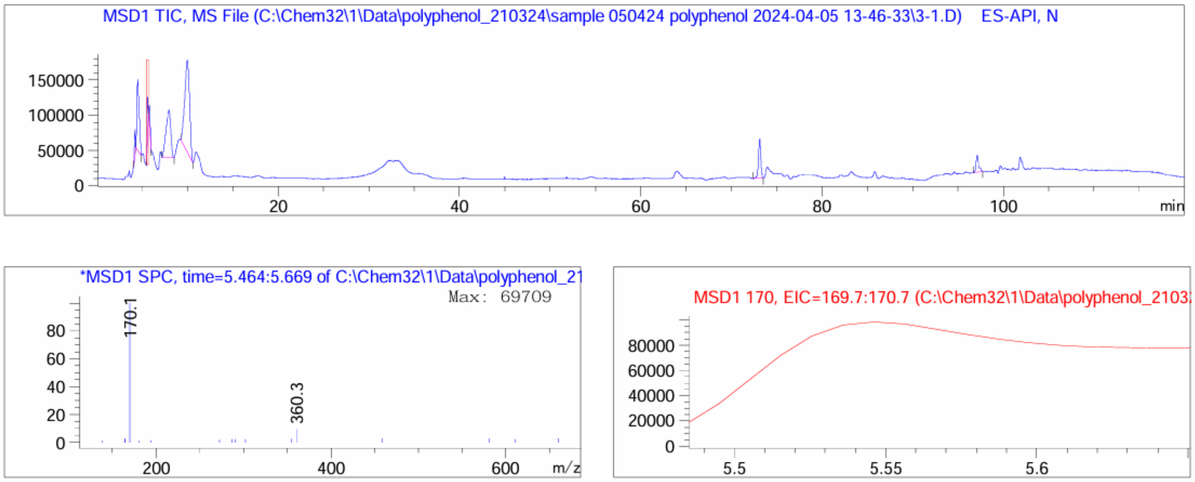


```
Peak #3 at  5.546 min ( 5.464 to  5.672 min)
-> The analysis found only one component, indicating a pure peak. <-

Component 1:  Peak at Scan 532.1. Top ions are  170
```

Figure S15. Mass spectral analysis of peak at retention time 5.546 min from C-3 extract.

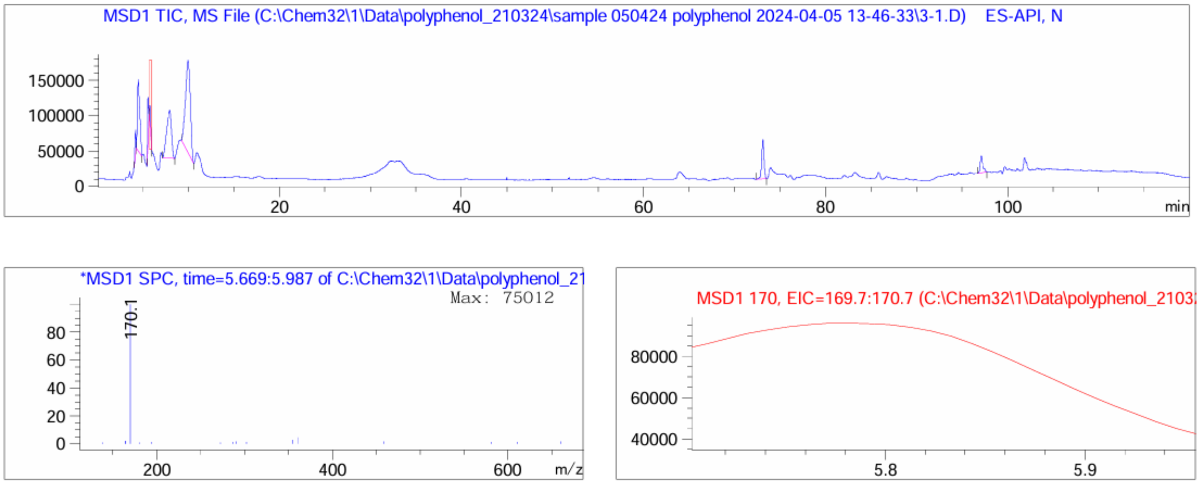


```
Peak #4 at  5.772 min ( 5.672 to  5.987 min)
-> The analysis found only one component, indicating a pure peak. <-

Component 1:  Peak at Scan 554.9. Top ions are  170
```

Figure S16. Mass spectral analysis of peak at retention time 5.772 min from C-3 extract.

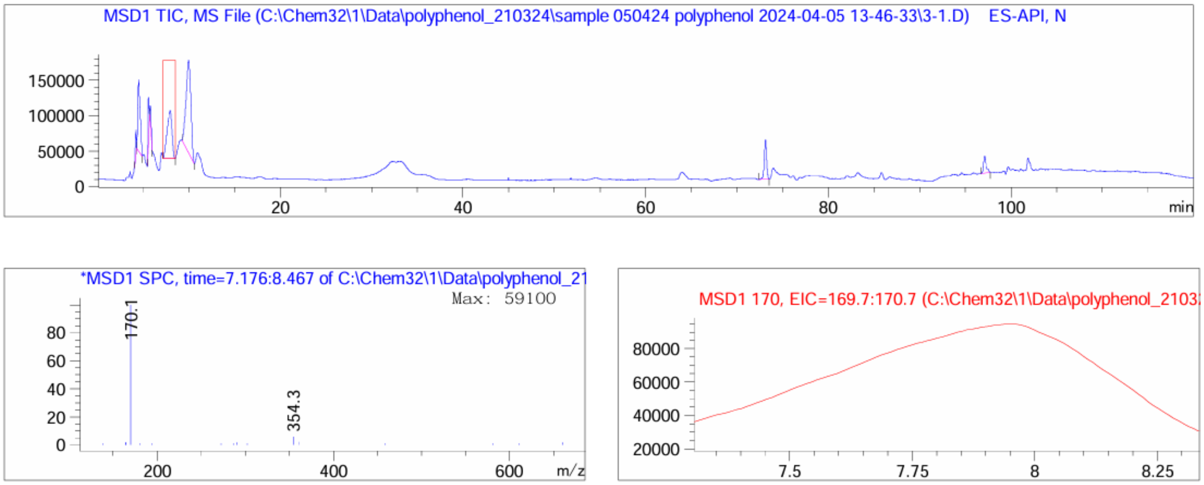


```
Peak #5 at  7.946 min ( 7.176 to  8.465 min)
-> The analysis found only one component, indicating a pure peak. <-

Component 1:  Peak at Scan 766.2. Top ions are  170
```

Figure S17. Mass spectral analysis of peak at retention time 7.946 min from C-3 extract.

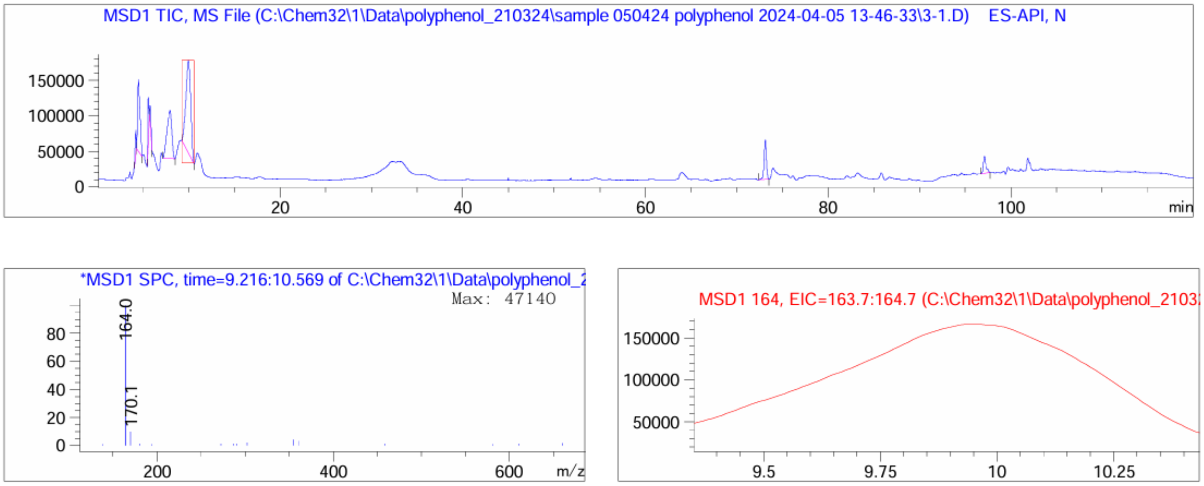

Peak #6 at 9.943 min (9.216 to 10.569 min)
-> The analysis found only one component, indicating a pure peak. <-

Component 1: Peak at Scan 961.1. Top ions are 164

Figure S18. Mass spectral analysis of peak at retention time 9.943 min from C-3 extract.

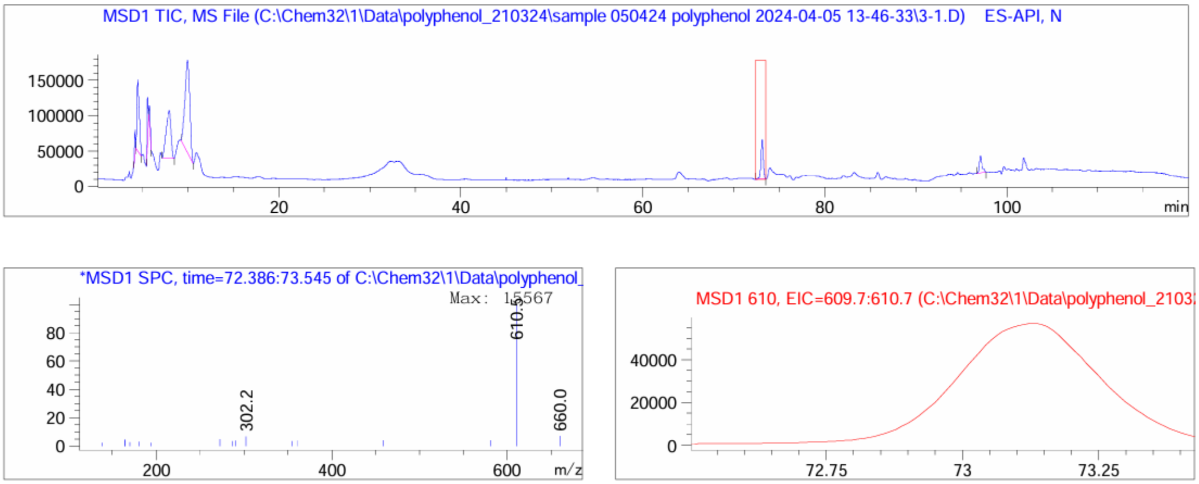

Peak #7 at 73.130 min (72.386 to 73.541 min)
-> The analysis found only one component, indicating a pure peak. <-

Component 1: Peak at Scan 7125.6. Top ions are 610

Figure S19. Mass spectral analysis of peak at retention time 73.130 min from C-3 extract.

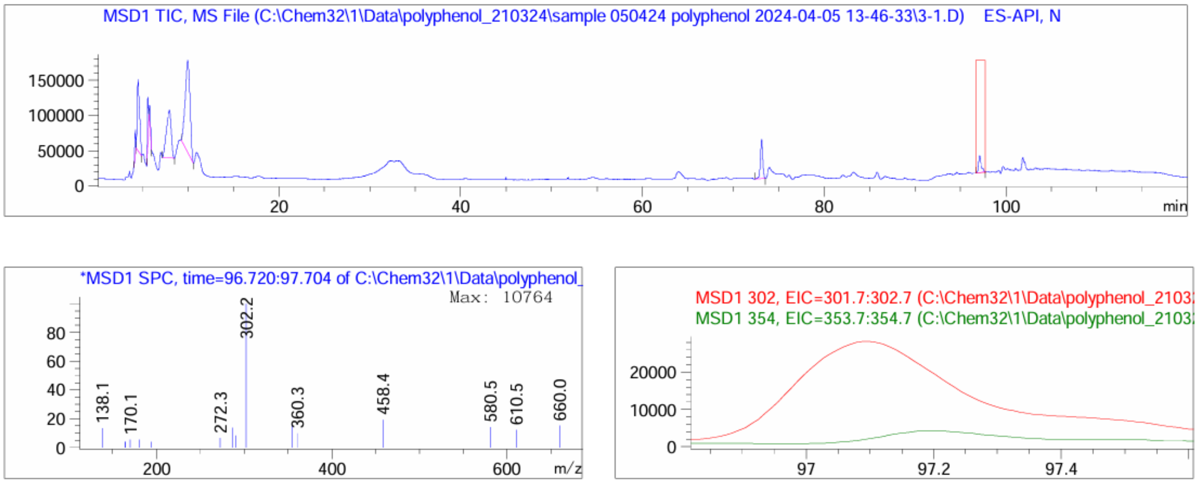


```
Peak #8 at 97.113 min (96.720 to 97.706 min)
-> The analysis found 2 components, indicating an impure peak. <-

Component 1:  Peak at Scan 9463.6. Top ions are  302
Component 2:  Peak at Scan 9473.6. Top ions are  354
```

Figure S20. Mass spectral analysis of peak at retention time 97.113 min from C-3 extract.

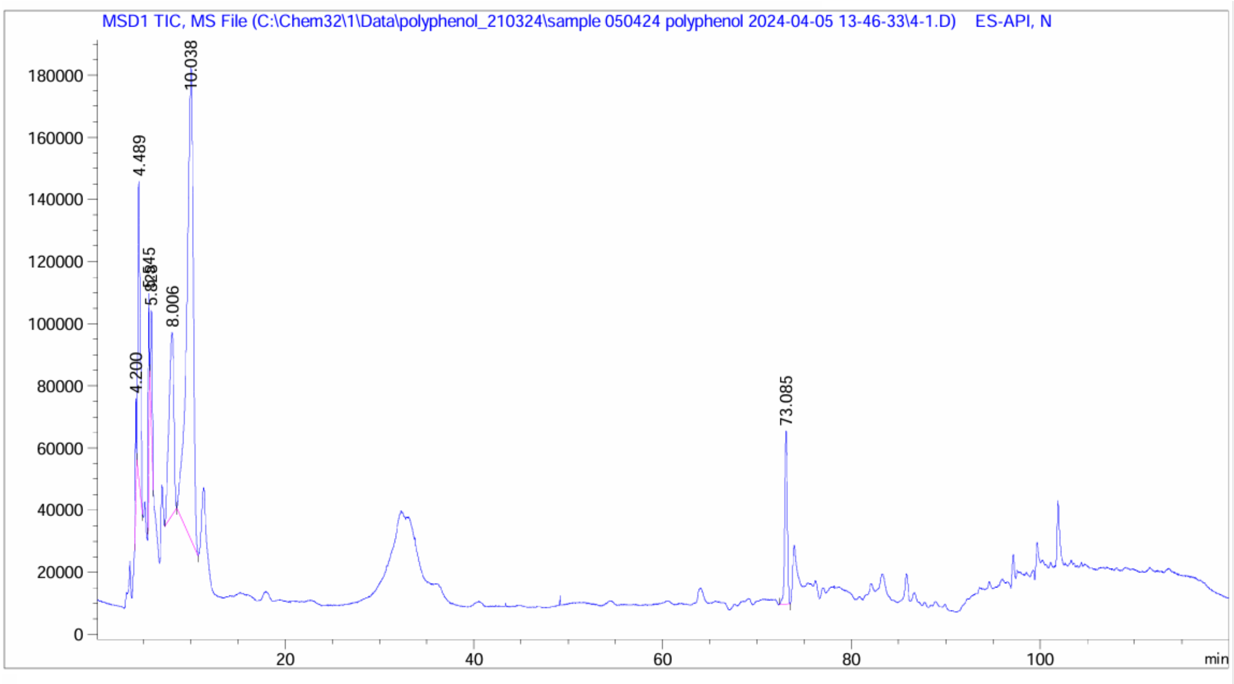

Figure S21. Total Ion Chromatogram (TIC) of C-4 extract analyzed by LC-MS in negative mode ESI, showing main peaks the main peaks of detected compounds.

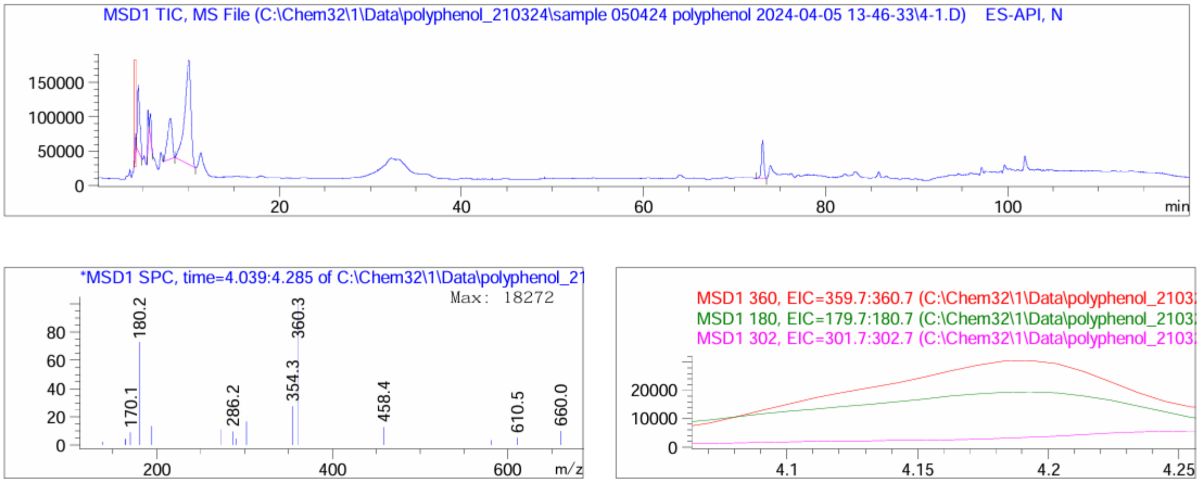

Peak #1 at 4.200 min (4.039 to 4.281 min)
-> The analysis found 2 components, indicating an impure peak. <-

Component 1: Peak at Scan 399.6. Top ions are 360 180 194
Component 2: Peak at Scan 405.8. Top ions are 302

Figure S22. Mass spectral analysis of peak at retention time 4.200 min from C-4 extract.

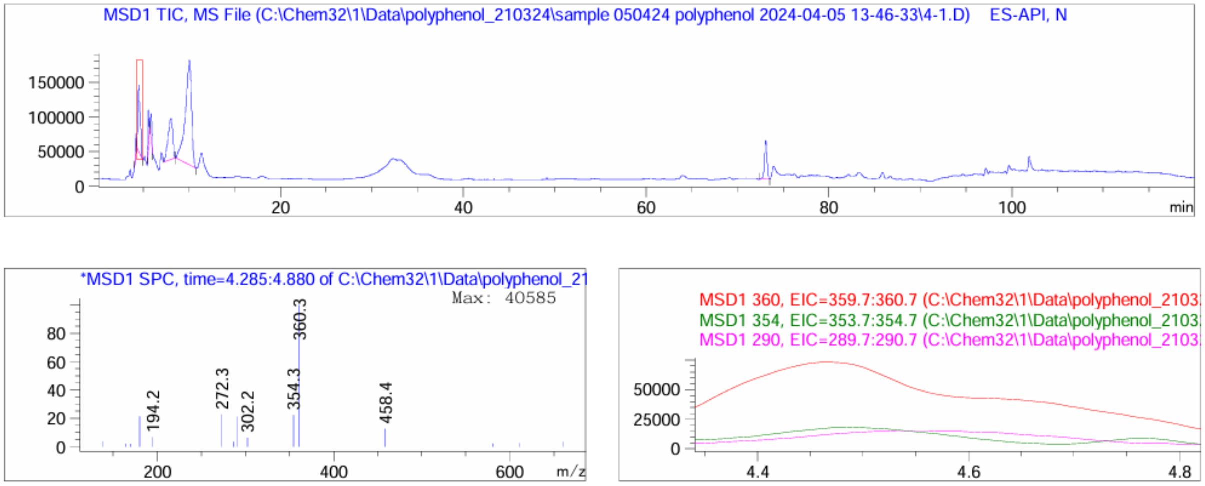


```
Peak #2 at  4.489 min ( 4.281 to  4.880 min)
-> The analysis found 5 components, indicating an impure peak. <-

Component 1:  Peak at Scan 426.8. Top ions are  360
Component 2:  Peak at Scan 428.8. Top ions are  354
Component 3:  Peak at Scan 434.5. Top ions are  290
Component 4:  Peak at Scan 437.0. Top ions are  272
Component 5:  Peak at Scan 456.2. Top ions are  180
```

Figure S23. Mass spectral analysis of peak at retention time 4.489 min from C-4 extract.

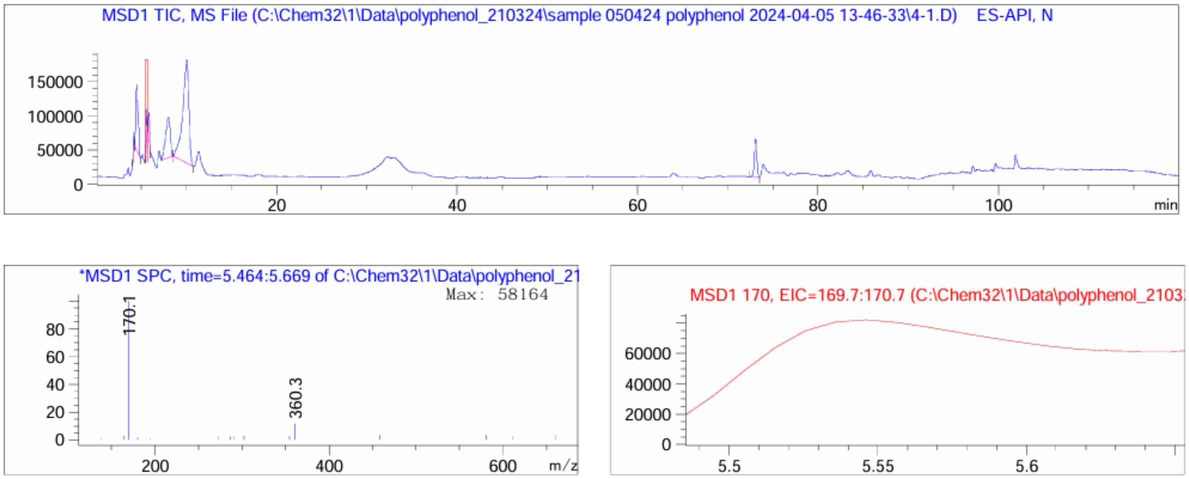


```
Peak #3 at  5.545 min ( 5.464 to  5.674 min)
-> The analysis found only one component, indicating a pure peak. <-

Component 1:  Peak at Scan 531.9. Top ions are  170
```

Figure S24. Mass spectral analysis of peak at retention time 5.545 min from C-4 extract.

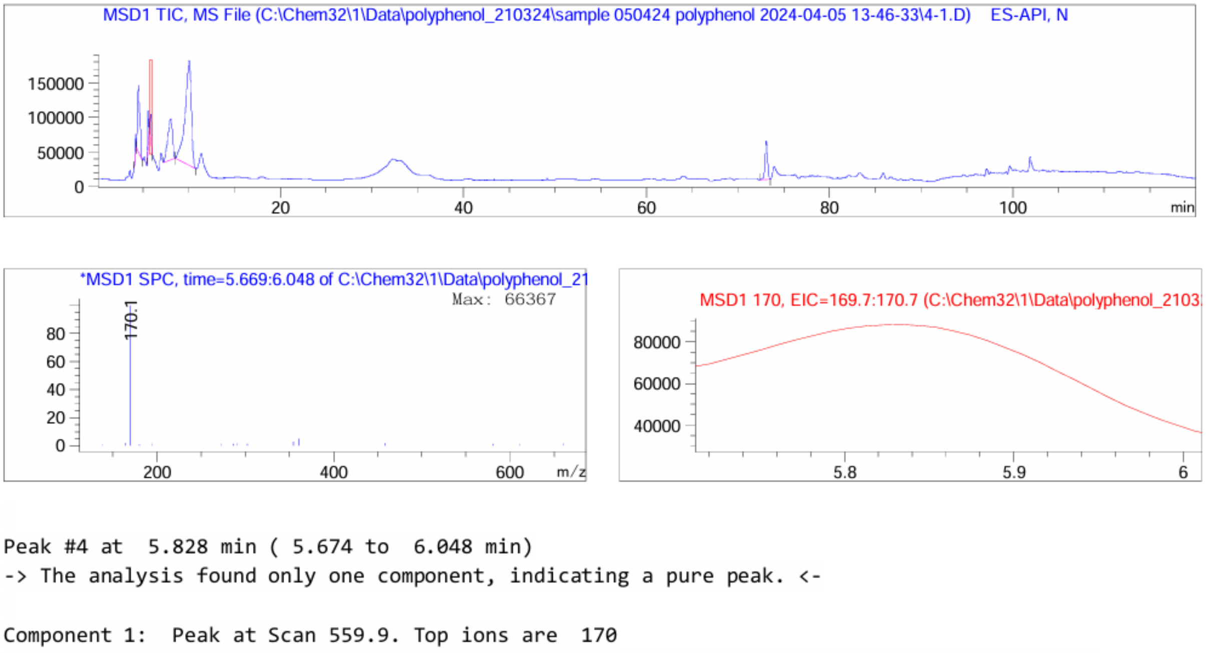

Figure S25. Mass spectral analysis of peak at retention time 5.828 min from C-4 extract.

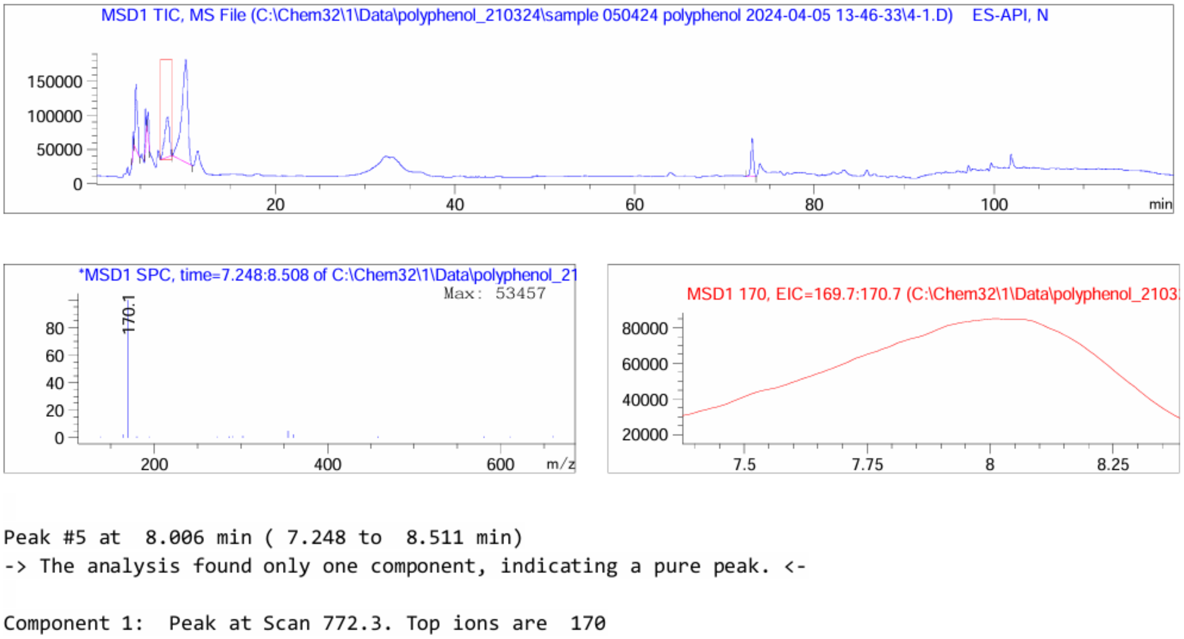

Figure S26. Mass spectral analysis of peak at retention time 8.006 min from C-4 extract.

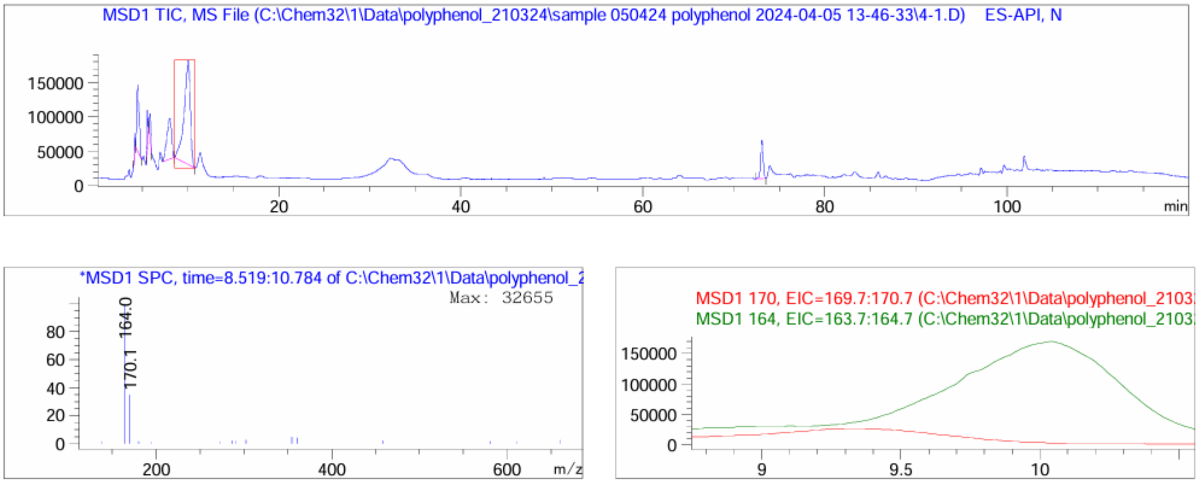


```
Peak #6 at 10.038 min ( 8.519 to 10.784 min)
-> The analysis found 2 components, indicating an impure peak. <-

Component 1:  Peak at Scan 901.4. Top ions are  170
Component 2:  Peak at Scan 970.4. Top ions are  164
```

Figure S27. Mass spectral analysis of peak at retention time 10.038 min from C-4 extract.

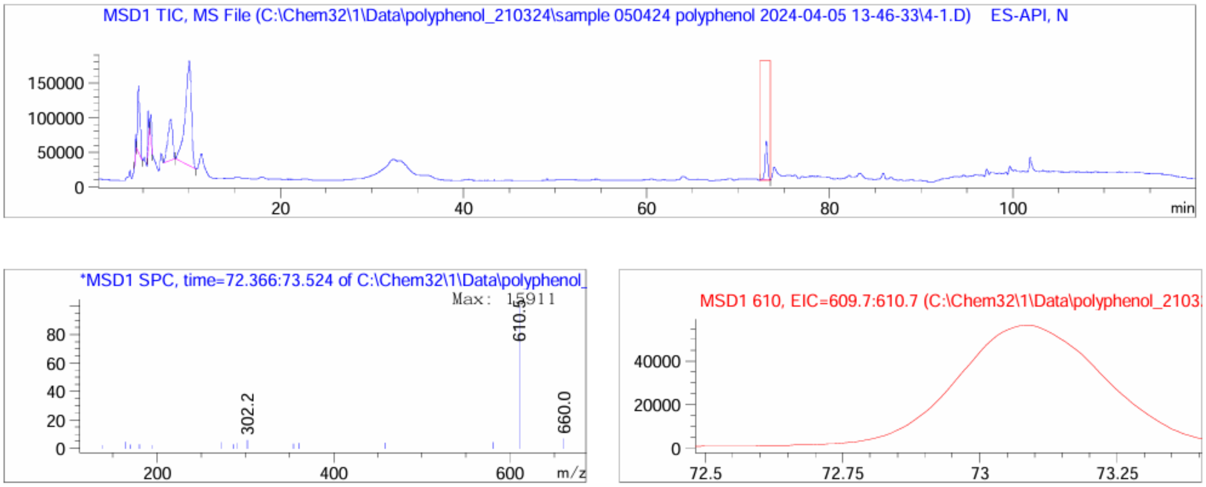


```
Peak #7 at 73.085 min (72.366 to 73.520 min)
-> The analysis found only one component, indicating a pure peak. <-

Component 1:  Peak at Scan 7121.3. Top ions are  610
```

Figure S28. Mass spectral analysis of peak at retention time 73.085 min from C-4 extract.
